# Genome-Wide Assessment for Genetic Variants Associated with Ventricular Dysfunction after Primary Coronary Artery Bypass Graft Surgery

**DOI:** 10.1371/journal.pone.0024593

**Published:** 2011-09-30

**Authors:** Amanda A. Fox, Mias Pretorius, Kuang-Yu Liu, Charles D. Collard, Tjorvi E. Perry, Stanton K. Shernan, Philip L. De Jager, David A. Hafler, Daniel S. Herman, Steven R. DePalma, Dan M. Roden, Jochen D. Muehlschlegel, Brian S. Donahue, Dawood Darbar, J. G. Seidman, Simon C. Body, Christine E. Seidman

**Affiliations:** 1 Department of Anesthesiology, Perioperative and Pain Medicine, Brigham and Women's Hospital, Harvard Medical School, Boston, Massachusetts, United States of America; 2 Department of Anesthesiology, Vanderbilt University School of Medicine, Nashville, Tennessee, United States of Ameica; 3 Division of Cardiovascular Anesthesia at the Texas Heart Institute, Saint Luke's Episcopal Hospital, Houston, Texas, United States of America; 4 Northwest Anesthesia PA, Minneapolis, Minnesota, United States of America; 5 Department of Neurology, Brigham and Women's Hospital, Harvard Medical School, Boston, Massachusetts, United States of America; 6 Department of Neurology, Yale School of Medicine, New Haven, Connecticut, United States of America; 7 Department of Genetics and Partners HealthCare Center for Genetics and Genomics, Harvard Medical School, Boston, Massachusetts, United States of America; 8 Departments of Medicine and Pharmacology, Vanderbilt University School of Medicine, Nashville, Tennessee, United States of America; 9 Department of Medicine, Vanderbilt University School of Medicine, Nashville, Tennessee, United States of America; 10 Division of Cardiology, Brigham and Women's Hospital, Harvard Medical School, Boston, Massachusetts, United States of America; 11 Howard Hughes Medical Institute, Boston, Massachusetts, United States of America; University of Colorado Denver, United States of America

## Abstract

**Background:**

Postoperative ventricular dysfunction (VnD) occurs in 9–20% of coronary artery bypass graft (CABG) surgical patients and is associated with increased postoperative morbidity and mortality. Understanding genetic causes of postoperative VnD should enhance patient risk stratification and improve treatment and prevention strategies. We aimed to determine if genetic variants associate with occurrence of in-hospital VnD after CABG surgery.

**Methods:**

A genome-wide association study identified single nucleotide polymorphisms (SNPs) associated with postoperative VnD in male subjects of European ancestry undergoing isolated primary CABG surgery with cardiopulmonary bypass. VnD was defined as the need for ≥2 inotropes or mechanical ventricular support after CABG surgery. Validated SNPs were assessed further in two replication CABG cohorts and meta-analysis was performed.

**Results:**

Over 100 SNPs were associated with VnD (P<10^−4^), with one SNP (rs17691914) encoded at 3p22.3 reaching genome-wide significance (P_additive model_ = 2.14×10^−8^). Meta-analysis of validation and replication study data for 17 SNPs identified three SNPs associated with increased risk for developing postoperative VnD after adjusting for clinical risk factors. These SNPs are located at 3p22.3 (rs17691914, OR_additive model_ = 2.01, P = 0.0002), 3p14.2 (rs17061085, OR_additive model_ = 1.70, P = 0.0001) and 11q23.2 (rs12279572, OR_recessive model_ = 2.19, P = 0.001).

**Conclusions:**

No SNPs were consistently associated with strong risk (OR_additive model_>2.1) of developing in-hospital VnD after CABG surgery. However, three genetic loci identified by meta-analysis were more modestly associated with development of postoperative VnD. Studies of larger cohorts to assess these loci as well as to define other genetic mechanisms and related biology that link genetic variants to postoperative ventricular dysfunction are warranted.

## Introduction

Each year almost 250,000 patients undergo coronary artery bypass graft (CABG) surgery in the United States alone, with the primary aim of surgery being to prevent major adverse cardiovascular events [1]. Despite the goal of improving health, 9–20% of patients undergoing cardiac surgery develop in-hospital postoperative ventricular dysfunction (VnD; defined as a new requirement for ≥2 inotropes or placement of an intra-aortic balloon pump or ventricular assist device), an outcome which is associated with significantly increased postoperative morbidity and mortality [2], [3], [4], [5]. Clinical predictors such as preoperative left ventricular ejection fraction (LVEF) and duration of cardiopulmonary bypass (CPB) explain only ∼20% of variability in occurrence of VnD after coronary artery bypass graft (CABG) surgery [6]. Given evidence for genetic influences on cardiac dysfunction, we therefore hypothesize that common genetic variants contribute to the development of postoperative VnD [6], [7].

We have previously examined candidate genetic loci and have identified single nucleotide polymorphisms (SNPs) within the *NPPA/NPPB* and *NPR3* natriuretic peptide system genes that associate with VnD after CABG surgery with adjusted odds ratios for association >2.0 [6]. However, to provide an unbiased assessment for common genetic variants that contribute strongly to this phenotype, we now have conducted an exploratory genome-wide association study (GWAS) of patients undergoing primary CABG surgery with CPB. From this GWAS we further assessed validated SNPs associated with VnD in two replication CABG cohorts and also conducted corresponding meta-analyses. Identification of novel genetic loci associated with VnD after CABG surgery provides the foundation from which to define causal genes and related molecular mechanisms. Such understanding may ultimately mitigate incidence of postoperative heart failure by suggesting new therapeutic opportunities for its prevention and treatment and by improving surgical risk stratification.

## Methods

### Study Subjects

Ethics Statement: In compliance with the Declaration of Helsinki, respective Institutional Review Board approval and subject written informed consent were obtained for all subjects (Partners Institutional Review Board, Boston, MA, USA; St. Luke's Episcopal Hospital Institutional Review Board, Houston, TX, USA; Vanderbilt University Institutional Review Board overseen by the Vanderbilt University Human Research Protection Program, Nashville, TN, USA).

Data were collected prospectively from primary CABG surgical patients at three institutions: Brigham and Women's Hospital (BWH; Boston, MA), the Texas Heart Institute (THI; Houston, TX), and Vanderbilt University Medical Center (Nashville, TN). These institutions enrolled subjects into two cardiac surgical cohorts: the CABG Genomics Program (BWH and THI) and the Vanderbilt Cardiac Surgery Registry (VCSR). De-identified control genotypes were provided for the GWAS from an ambulatory multiple sclerosis study cohort of European ancestry subjects. Information S1 further describes the study cohorts.

CABG subjects were prospectively excluded from analyses if they had emergency surgery, re-operative cardiac surgery, concurrent valve surgery, no aortic cross-clamp, or CPB >220 minutes, or if preoperatively they had moderate to severe mitral regurgitation, LVEF <20%, severe preoperative renal dysfunction (hemodialysis or serum creatinine >3 mg/dL), or were receiving inotrope, intra-aortic balloon pump or ventricular assist device support. To minimize false positive SNP associations related to population stratification, all analyses were restricted to European ancestry subjects. With the exception of the GWAS' controls, analyses did not include women, as gender may influence development of heart failure, and the number of female subjects with VnD enrolled in the CABG Genomics and VCSR cohorts (<20%) was insufficient to support gender stratified SNP association analyses [5], [8], [9].

Postoperative VnD cases were identified in accordance with prior studies [3], [6], [10], with the VnD phenotype defined as a new requirement for ≥2 inotropes, or new placement of an intra-aortic balloon pump or ventricular assist device either during the intraoperative period after the patient separated from CPB or postoperatively in the intensive care unit. Since postoperative intensive care unit data was not obtained for VCSR subjects, VnD was assessed using intra-operative criteria for this cohort. Inotrope support was defined as continuous infusion of amrinone, milrinone, dobutamine, dopamine (>5 mcg/kg/min), epinephrine, isoproterenol, norepinephrine, or vasopressin.

### Genotyping

#### Genomic Arrays

Genotypes were ascertained using the Affymetrix 6.0 Genome-Wide Human SNP Array (Affymetrix, Santa Clara, CA) and were analyzed using the Birdseed (version 2) algorithm. SNPs were excluded from analyses for call rates <95%, minor allele frequencies <1%, or for not exhibiting Hardy Weinberg equilibrium (P<10^−6^). SNPs were also excluded if they had a plate association (P<10^−10^) or differential missingness either between cases and controls (P<0.001) or based on flanking haplotypes (P<10^−10^; flanking haplotypes consisted of the 2 SNPs that were the closest adjacent Array SNPs upstream and downstream of the assessed SNP).

#### Individual SNP Genotypes

Genotypes were determined using Sequenom MassArray iPLEX® (Sequenom, San Diego, CA) and the manufacturer's protocols. Sequenom raw data were analyzed with the SpectroTyper 3.4 software (Sequenom, San Diego, CA). Spectra and cluster plots were checked by visual inspection of intensity plots with manual curation of genotype calls.

### Statistical Analyses

#### Genome-Wide Association Study

Subjects were excluded from GWAS analyses if they did not self-identify to be of European ancestry. In addition, population stratification outliers were excluded from GWAS analyses as described in Information S1, and multidimensional scaling analysis was used to provide further reassurance that population stratification did not influence GWAS association results [11]. GWAS data was assessed using PLINK (version 1.07) [11].

Because the cohort size of this exploratory study would detect only genetic variants with strong associations with development of postoperative VnD, a Type 1 error rate <0.0005 was utilized for statistical power assessments. This P value threshold was chosen with the understanding that a proportion of SNP associations detected at this level of significance could be false positive associations and that further assessment would be conducted beyond the initial GWAS. Based on the available case to control ratio of 1∶14 and assuming an additive genetic model, 12% incidence of VnD, 80% power, minimum estimated detectable genetic risk ratios for the GWAS were 2.1, 2.2, 2.3, 2.5, and 2.8 for SNPs with minor allele frequencies of >0.25, 0.25, 0.20, 0.15, and 0.10 respectively [12]. Consequently, SNPs with minor allele frequencies <5% were not analyzed for association with VnD.

Association between SNPs and VnD were estimated using the trend test for the additive genetic model, the Pearson chi-square test for allelic and dominant genetic models, and the Fisher's Exact test for the recessive genetic model. Using genomic inflation factor lambdas genomic-control corrections were made to account for residual population stratification effects suggested by Q-Q plots (Figure S1). Cluster plots (Figure S2) for SNPs associated with VnD were visually inspected and SNPs with poor differentiation of heterozygote and homozygote calls were eliminated.

#### Validation and Replication Studies

Categorical and continuous patient demographic and clinical characteristics were compared between case and control groups for the CABG Genomics validation study, Vanderbilt replication study, and CABG Genomics replication study using Pearson's chi-squared, Fisher's Exact or student's t-tests where appropriate (JMP version 7.0.1, SAS Institute, Cary, NC). PLINK (version 1.07) was used for genetic analyses [11]. Hardy Weinberg Equilibrium was evaluated using Fisher's Exact tests.

Univariate SNP associations with VnD were assessed using Pearson chi-square tests for the allelic model and logistic regression for the additive, dominant and recessive models. SNP associations (additive, dominant and recessive models) were additionally adjusted for age, LVEF, CPB time, and institution using logistic regression. Univariate and multivariable adjusted odds ratios (OR) and 95% confidence intervals (CI) were determined for each SNP. Point-wise permutation analyses (10,000 rounds of permuting case/control status for each genetic model) were conducted for each SNP (univariate and multivariable covariate adjusted assessments). In order to assess that observed SNP associations with VnD were not a consequence of associations with presenting LVEF, linear regression was used to conduct univariate assessments of CABG Genomics validation study SNPs for association with preoperative LVEF. Meta-analyses were conducted for SNPs successfully genotyped for both replication studies (methods described in Information S1). Bonferroni adjustments were applied to analyses to correct for multiple SNP tests.

## Results

**Figure 1:**
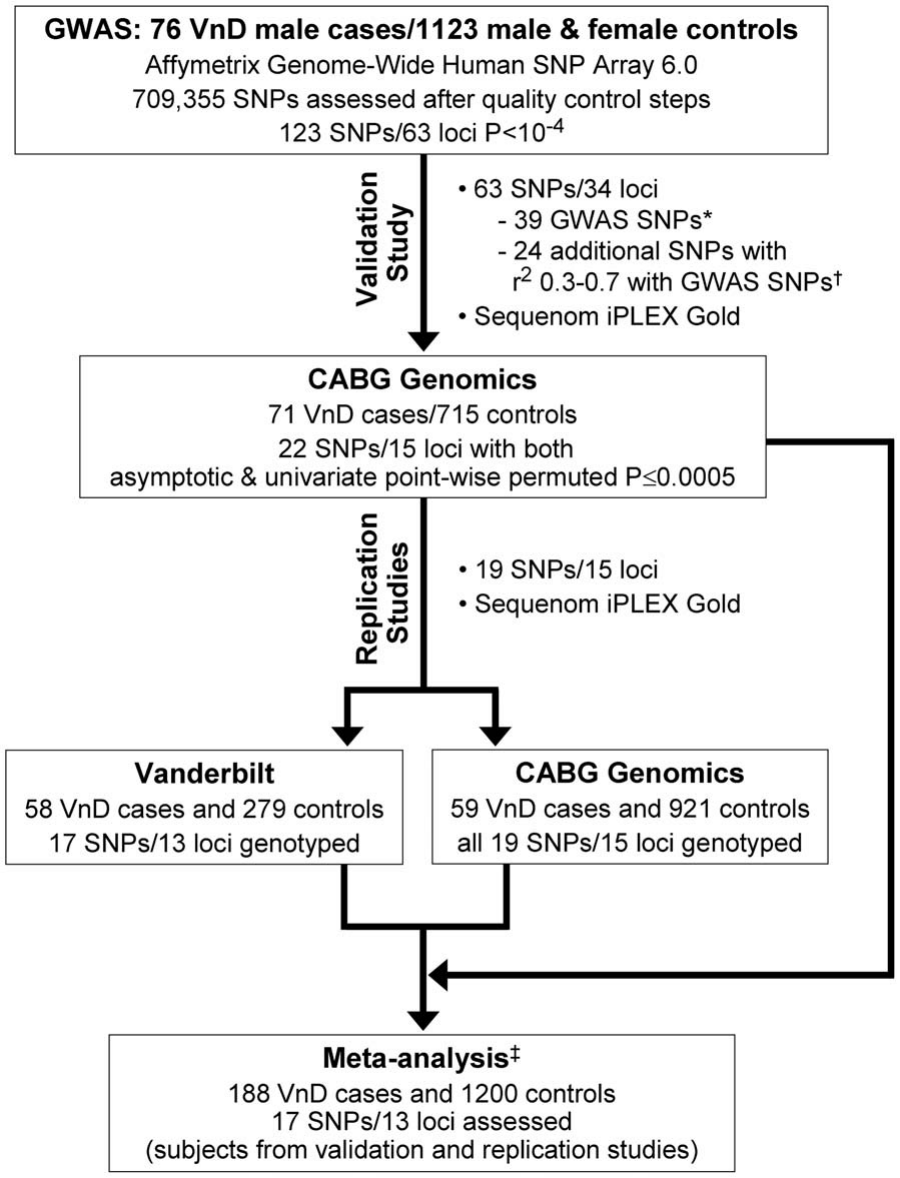
Schematic illustrating sequence of GWAS study followed by validation study, replication studies, and meta-analysis. All subjects were of European ancestry and were male unless otherwise specified. * 39 Affymetrix 6.0 SNPs associated with VnD in the GWAS (P<10^−4^) or SNPs in strong linkage disequilibrium (r^2^>0.80; estimated using HaploView 4.1.) with these GWAS SNPs were genotyped from 34 genetic loci for further validation study assessment. † In order to identify SNPs with potentially stronger associations with VnD within some of the genetic loci identified in the GWAS, additional SNPs were genotyped that were in moderate linkage disequilibrium with associated GWAS SNPs (r^2^ = 0.30–0.70). Correlations (r^2^) were estimated using HaploView 4.1. ^‡^ SNP associations with VnD were analyzed by enrolling institution (BWH, THI, and Vanderbilt University Medical Center) with adjustments for age, preoperative LVEF, and duration of CPB time, and then combined by meta-analysis.

We performed a GWAS study that compared CABG Genomics cases with ambulatory control subjects. Validated SNPs associated with VnD in the GWAS study were further assessed using CABG Genomics control subjects. SNPs from this analysis were then evaluated in replication studies of the CABG Genomics and VCSR cohorts, and related meta-analyses were performed (Figure 1).

Demographic and clinical characteristics for the study cohorts are shown in Table 1. Although gender was the only demographic information available for the multiple sclerosis study cohort, these subjects only provided the control genotype data used in the GWAS analyses.

**Table 1:** Study cohort characteristics of cases and controls undergoing primary coronary artery bypass grafting with cardiopulmonary bypass.

Subject Group (number of subjects)	Age years	BMI kg/m^2^	Diabetes	Hypertension	Smoking >30 pyh	LVEF %	CPB time minutes
CABG Genomics validation cases (n = 71)*	65±11	30±5	35.2%	74.6%	45.5%‡	42±14‡ §	112±34‡
CABG Genomics validation controls (n = 715)†	64±10	30±5	25.9%	72.7%	29.6%‡	54±11‡	93±34‡
CABG Genomics replication cases (n = 59)	64±9	30±7	30.5%	71.2%	38.5%	49±15‡ §	102±37‡
CABG Genomics replication controls (n = 921)†	63±10	30±6	26.3%	73.9%	29.3%	54±11‡	92±35‡
Vanderbilt replication cases (n = 58)	63±11	29±5	38.6%	71.9%	46.6%	35±13‡ §	113±34‡
Vanderbilt replication controls (n = 279)	61±10	29±5	32.0%	74.9%	39.9%	48±8‡	110±30‡

Data shown as (%) for dichotomous variables and mean ± standard deviation for continuous variables.

BMI = body mass index; CPB = cardiopulmonary bypass; LVEF = left ventricular ejection fraction; pyh = pack year history; SNP = single nucleotide polymorphism.

*Genome-wide association study assessed 76 ventricular dysfunction cases; subsequent SNP analyses were successfully validated for 71 of these ventricular dysfunction cases.

†CABG Genomics replication controls include control subjects used in the CABG Genomics validation study.

‡ Significantly different between cases and controls (P<0.05).

§ Significantly different than other two case groups (P<0.05).

As expected there were significant differences in the clinical characteristics of CABG subjects with and without VnD (cases and controls, respectively). All VnD cases had significantly lower preoperative LVEFs and longer CPB times than CABG controls (P<0.05), and CABG Genomics cases used for validation had more subjects with a >30 pack year history of smoking than CABG controls (P<0.05). VnD cases assessed in validation and replication studies differed significantly between groups with regards to preoperative LVEF, but not other characteristics.

### Genome-Wide Association Study

We surveyed the genomes of 92 male VnD cases using Affymetrix 6.0 arrays. Sixteen samples were subsequently excluded from analysis based upon genotyping quality control, gender mismatch, population stratification outliers, and cryptic relatedness (methods described in Information S1). Applying these same filters, 1123 ambulatory controls (28% male) were appropriate to be studied from the multiple sclerosis study cohort. Based on these cohort sizes, this GWAS was powered to detect genetic risk ratios ≥2.1.

**Figure 2:**
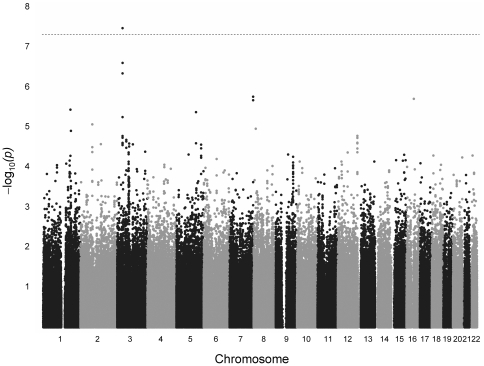
The –log P value (lambda adjusted) of the allelic genetic model for each single nucleotide polymorphism (SNP) according to location on the 22 autosomal chromosomes. Horizontal line indicates the 5×10^−8^ P value threshold for genome-wide significance.

Analyses of 709,355 SNPs identified 123 SNPs (63 genetic loci) that were associated with VnD with P values <10^−4^ (Table S1). One SNP, rs17691914, reached genome-wide significance by allelic (P = 3.4×10^−8^) and additive models (P = 2.1×10^−8^). This SNP had minor allele frequencies of 21% and 7% in VnD cases versus controls, respectively. An additional 10 SNPs associated with VnD with P values <10^−5^. Three of these 10 SNPs (rs9835451, rs1979406, and rs17032625) reside within the same chromosome 3 locus as rs17691914 (chromosome 3∶ 34,902,008–35,052,013 bp). Of the SNPs with P values <10^−5^, rs2171325 was not assessed in further validation or replication studies because the minor allele frequency was <10% in both cases and controls. Figure 2 shows allelic model results for association with VnD across all 22 autosomes.

### CABG Genomics Technical Validation and CABG Control Studies

GWAS SNPs with a minor allele frequency>10% and P<10^−4^ for association with VnD (after genomic-control corrections for allelic, additive, dominant and recessive genetic models) were selected for further assessment in CABG Genomics cases and controls. Five samples that failed ≥90% of validation study genotyping and one SNP (rs11060480) that did not technically validate were excluded from further study. After clinical and genotyping exclusions, data for 71 of the 76 GWAS VnD cases and 715 male CABG controls (subjects without VnD) were analyzed. The technical validation aspect of this study compared minor allele frequencies for the VnD cases for Affymetrix 6.0 versus Sequenom genotyping in order to identify potential false positive GWAS associations due to technical calling errors. In addition, this study refined the initial genetic association study in order to: 1) assess if GWAS associations persisted when the VnD case data was compared to male CABG control data; 2) allow assessment of additional SNPs with moderate linkage disequilibrium with identified GWAS SNPs (correlation r^2^ with GWAS SNPs = 0.30–0.70) in order to identify SNPs within GWAS loci with potentially stronger associations with VnD; 3) allow covariate adjustment of SNP associations for age, institution, LVEF and duration of CPB; 4) allow assessment of SNP associations with preoperative LVEF.

Sixty-three SNPs (36 GWAS SNPs and 27 SNPs in linkage disequilibrium with these SNPs) were assessed in 71 VnD cases and 715 CABG controls (Table S2). Twenty-two SNPs encoded at 15 loci had both asymptotic and point-wise permuted univariate P values ≤0.0005, and we selected 19 of these SNPs for further exploration in replication studies (Table 2). After Bonferroni correction, none of these SNPs showed significant association with preoperative LVEF, the primary clinical predictor of VnD [6].

**Table 2:** CABG Genomics validation study: Nineteen validated SNPs associated with ventricular dysfunction after primary coronary artery bypass grafting with cardiopulmonary bypass.*

SNP	Chromosome, Location NCBI hg36	Gene	GWAS or Additional SNP	Minor Allele/Major Allele	MAF VnD Cases (n = 71)/Controls (n = 715)	Best Genetic Model in GWAS†	Unadjusted Odds Ratio	Univariate Asymptotic P value	Multivariable Adjusted Odds Ratio	Multivariate Asymptotic P value
**rs1287820**	Chr 1, 180,855,954		GWAS	G/C	0.32/0.20	Additive	2.00	0.0004	2.26	0.0002
**rs17691914**	Chr 3, 34,937,807		GWAS	G/A	0.19/0.08	Additive	2.74	2.7×10^−5^	2.96	6.0×10^−5^
**rs9835451**	Chr 3, 34,946,568		GWAS	G/A	0.22/0.11	Additive	2.14	0.0005	2.29	0.001
**rs17358517**	Chr 3, 59,660,772		GWAS	T/C	0.38/0.23	Additive	2.07	9.6×10^−5^	2.14	0.0002
**rs17061085**	Chr 3, 59,666,765		Additional	A/G	0.26/0.13	Additive	2.04	0.0001	2.08	0.0004
**rs9837024**	Chr 3, 78,534,327		GWAS	G/A	0.36/0.22	Additive	2.09	0.0002	1.95	0.002
**rs4242051**	Chr 5, 54,234,532		GWAS	T/C	0.40/0.28	Recessive	3.30	0.0002	2.73	0.005
**rs6459959**	Chr 7, 155,390,912		GWAS	C/G	0.51/0.36	Additive	1.88	0.0004	1.78	0.002
**rs6459961**	Chr 7, 155,391,016		GWAS	C/A	0.51/0.36	Additive	1.88	0.0004	1.78	0.002
**rs10104640**	Chr 8, 40,762,563	*ZMAT4*	GWAS	A/C	0.38/0.27	Recessive	3.43	6.7×10^−5^	3.51	0.0003
**rs10500830**	Chr 11, 16,441,827	*SOX6*	GWAS	A/G	0.28/0.19	Recessive	5.37′	3.6×10^−5^	6.18	0.0001
**rs12279572**	Chr 11, 117,458,170	*TMPRSS4*	GWAS	G/A	0.36/0.29	Recessive	3.61	3.5×10^−5^	3.61	0.0002
**rs7975290**	Chr 12, 26,505,185	*ITPR2*	GWAS	G/A	0.14/0.05	Additive	2.91	8.4×10^−5^	3.53	4.5×10^−5^
**rs10773689**	Chr 12, 128,686,079	*TMEM132D*	GWAS	A/C	0.24/0.39	Additive	0.50	0.0007	0.47	0.0005
**rs10519861**	Chr 15, 31,773,350	*RYR3*	GWAS	T/G	0.56/0.40	Additive	2.00	0.0001	2.18	0.0001
**rs8027394**	Chr 15, 76,404,968		GWAS	T/C	0.48/0.30	Additive	2.06	4.2×10^−5^	2.12	0.0002
**rs12593362**	Chr 15, 76,421,786		Additional	T/G	0.47/0.31	Additive	1.99	0.0001	2.10	0.0002
**rs8058644**	Chr 16, 48,454,181		GWAS	T/C	0.18/0.07	Additive	2.61	6.8×10^−5^	2.86	8.7×10^−5^
**rs16974035**	Chr 18, 10,269,356		GWAS	G/A	0.49/0.34	Additive	1.86	0.0005	1.96	0.0005

Chr = chromosome; GWAS = genome-wide association study; MAF = minor allele frequency; SNP = single nucleotide polymorphism; VnD = ventricular dysfunction.

*19 SNPs with univariate asymptotic and point-wise permuted P values≤0.0005 (allelic, additive, or recessive models) were selected from the CABG Genomics validation study's 63 assessed SNPs (Table S2) for further assessment.

†When GWAS data indicated allelic genetic model as the most significant, Table 2 reports additive model results for the validation study to show multivariate adjusted results.

### Replication Studies

The 19 SNPs selected for replication were assessed initially in the VCSR cohort. Two SNPs, rs9837024 and rs10773689, were excluded due to failed genotyping and Hardy Weinberg Equilibrium testing in controls, respectively. Results for the remaining 17 SNPs (encoded at 13 loci) are shown in Table S3. For 10 SNPs (encoded at 7 loci) the direction of the ORs was consistent across the GWAS, validation, and VCSR replication studies, but only rs17061085 associated with VnD with P<0.05 (unadjusted OR_additive model_ = 1.65, P = 0.04; covariate adjusted OR_additive model_ = 1.69, P = 0.056). This association is not significant after Bonferroni adjustment.

We also assessed replication in the CABG Genomics cohort using previously unstudied cases. All 19 SNPs were successfully genotyped, and for 12 SNPs (encoded at 10 loci), the direction of the ORs was consistent across the GWAS and validation studies. However, none of these 12 SNPs achieved significant association with VnD (P<0.05) in univariate or multivariable adjusted analyses (Table S4).

### Meta-analyses

To assess for more modest risk (OR <2.1) that might be associated with putative VnD SNPs, we performed a meta-analysis of the VCSR and CABG Genomics cohort replication studies. SNP rs17061085 was associated with postoperative VnD with the same direction of effect as the GWAS, even after adjusting for age, preoperative LVEF and CPB time (OR = 1.44, P = 0.04, Cochrane Q statistic test for heterogeneity P = 0.47 and I^2^ heterogeneity index = 0). However, this association does not achieve statistical significance after Bonferroni adjustment.

In order to identify SNPs with the strongest associations with VnD within the overall group of CABG subjects assessed in this study, we also pooled all VnD case (n = 188) and non-VnD CABG control (n = 1200) data from the validation and replication studies using meta-analysis (Table S5). Table 3 shows the three SNPs (rs17691914, rs17061085, and rs12279572) having the smallest P values (P≤0.001) for association with VnD after adjusting for age, preoperative LVEF and duration of CPB.

**Table 3:** Most significant SNP associations with ventricular dysfunction after primary coronary artery bypass grafting with cardiopulmonary bypass in covariate adjusted meta-analyses.*

SNP	MAF VnD Cases (n = 188)/MAF Controls (n = 1200)	Genetic Model†	Odds Ratio‡	P value‡	Heterogeneity P value§	Genes within 250 kb either upstream or downstream of SNP location
rs17691914	13.8%/8.2%	Additive	2.01	0.0002	0.64	None
		Dominant	2.20	8.1×10^−5^	0.50	
rs17061085	20.2%/12.0%	Additive	1.70	0.0001	0.72	*FHIT, LOC339902*
rs12279572	32.2%/29.1%	Recessive	2.19	0.001	0.61	*FXYD6, TMPRSS13, IL1ORA, TMPRSS4, SCN4B, SCN2B, AMICA1, LOC100128245, LOC100129498, LOC100131626, MPZL2, MPZL3, CD3E*

MAF = minor allele frequency; SNP = single nucleotide polymorphism; VnD = ventricular dysfunction;

*Cases and controls were from the CABG Genomics validation and replication studies and the Vanderbilt replication study. SNP data for these subjects were assessed by institution, with covariate adjustments made for age, preoperative left ventricular ejection fraction, and duration of cardiopulmonary bypass. Results were then combined using meta-analysis. 17 SNPs studied across the CABG Genomics validation study and the Vanderbilt and CABG Genomics replication studies were assessed. Meta-analysis results for all 17 SNPs are shown in Table S5.

†Results for best genetic models in the genome-wide association study (additive, dominant, or recessive).

‡ Odds ratios and P values derived using random effects meta-analysis.

§ P value for Cochran's Q statistic assessment of heterogeneity (P<0.05 would mean effect sizes between institutions are significantly different). The I^2^ heterogeneity indices equaled 0 for all three SNPs shown in the Table.

## Discussion

VnD after cardiac surgery is associated with significant patient morbidity and mortality. Although clinical parameters such as preoperative LVEF and duration of CPB are predictive of risk for developing postoperative VnD [3], [5], other unknown factors are expected to contribute to postoperative cardiac dysfunction. Using genome-wide assessments, we defined 3 novel genetic variants (rs17691914 on 3p22.3; rs17061085 on 3p14.2; rs12279572 on 11q23.2) that associate with development of VnD after primary CABG surgery, even after adjusting for age, preoperative LVEF, and prolonged CPB time. These data suggest genetic risk as a contributor to cardiac dysfunction that occurs in the setting of CABG and presumably other overt myocardial stresses.

Future study of the genes at the 3 loci tagged by these VnD-associated SNPs may enhance understanding of the pathophysiology of postoperative VnD. The 3p22.3 locus (defined by rs17691914) is located within 1 Mb of the programmed cell death 6-interacting protein gene (*PDCD6IP*). Despite a paucity of other genes within close proximity to rs17691914, we are intrigued that this SNP achieved genome-wide significance in the initial GWAS and that the 3p22.3 is also tagged by a SNP (rs12638540) within an intron of the CKLF-like MARVEL transmembrane domain containing 7 gene (*CMTM7*) that is reported to be significantly associated with heart failure mortality (Hazard Ratio = 1.53; P = 3.21×10^−7^) in a GWAS meta-analysis of 2,526 ambulatory patients [13]. An interesting possibility is that these 3p22.3 SNPs are linked to a single causal gene that impacts cardiac function.

SNP rs17061085, which maps to 3p14.2, resides near the fragile histidine triad (*FHIT*) gene that encodes a protein implicated in multiple cancers [14]. While *FHIT* has not been well studied in cardiac tissue, human atrial expression of *FHIT* is diminished in the setting of decreased LVEF [15].

The 11p11.2 intronic SNP rs12279572 lies within the *TMPRSS4* (transmembrane protease, serine 4 isoform-1) gene that encodes a protein implicated in facilitating the invasion, migration and metastasis of human tumor cells via epithelial-mesenchymal transitions [16]. This protein has unknown relevance to cardiac biology. Twelve other known genes are encoded within 250 kbps of SNP rs12279572. These include genes encoding interleukin-10 receptor A (*IL10RA*), which may participate in the oxidative-stress responses that promote cardiac remodeling and heart failure [17], and 2 sodium channel subunits (*SCN4B* and *SCN2B*) that are expressed in cardiac myocytes and are mutated in long-QT syndrome and atrial fibrillation, respectively [18], [19].

### Limitations

We recognize several important limitations of our study, including the small size of the study cohorts and the narrow demographic profiles we were consequently able to examine. We consider this study to be an exploratory assessment for strong genetic influences on the postoperative ventricular dysfunction outcome, as cohort size restricted our ability to detect SNPs associated with VnD with genetic risk ratios <2.1 for an additive genetic model. We secondarily assessed dominant and recessive genetic models, albeit with statistical power to detect substantially higher genetic risk ratios for these models than for the additive model. Our findings have important implications for design of future studies, in that no SNPs consistently associated with postoperative VnD with additive model ORs >2.1. However, GWASs of cardiovascular disease in very large non-surgical populations illustrate the potential to discover SNPs associated with more modest risks of developing common disease. For example, loci found to be associated with coronary artery disease and/or myocardial infarction [20], [21], [22], [23] in large ambulatory GWAS assessments reported lower ORs (additive model ORs of 1.10–1.65) than those achieved here. As such we expect that assessments of postoperative VnD in larger surgical cohorts will be needed to define loci with more modest associations with this phenotype.

We recognize that gaps in the Affymetrix 6.0 Genome-Wide array may still potentially miss important SNP associations with VnD, as all commercially available genome-wide SNP arrays have some gaps in coverage of genetic variability across the genome [24]. For example, we previously identified SNPs within the *NPPA/NPPB* gene complex on chromosome 1 that significantly associate with VnD after CABG surgery (additive model ORs 1.85–2.29)[6]; yet, these *NPPA/NPPB* SNPs were not assessed in the Affymetrix 6.0 SNP array platform. However, the Affymetrix 6.0 Genome-Wide array is estimated to cover ∼83% of genome-wide SNP variability, thus providing very good potential for this study to detect novel SNP associations with VnD after CABG surgery [24].

Another important limitation of this study was the narrow demographic profile of the analyzed study cohorts. There were not enough women enrolled within the CABG Genomics and VCSR cohorts to allow for SNP association analyses stratified by gender. Nor were there sufficient non-European ancestry subjects of either gender to allow for SNP association analyses stratified by ethnicity. Limiting this study to men of European ancestry enhanced homogeneity of the study cohort for the purpose of best allowing detection of real SNP associations with VnD that were not confounded by influence of gender and racial background. However, we therefore propose that further evaluations of VnD-associated SNPs be conducted in women and in both men and women of non-European ancestry when cohorts are available that contain sufficient sample sizes of these populations.

### Conclusions

In conclusion, at least 3 loci identified by genome-wide and subsequent validation and replication assessments were associated with modest risk for VnD after primary CABG surgery. These and other SNPs within the surrounding genetic loci warrant further replication in large cardiac surgical cohorts. With continued identification of genotypes associated with postoperative VnD, we expect to improve risk stratification and gain insights into biologic pathways linking these variants to postoperative myocardial dysfunction. Such knowledge could lead to new preventions and therapies for postoperative VnD, and, possibly other forms of heart failure.

## Supporting Information

Figure S1 **Genome wide association study Q-Q plots of the expected and observed –log P values for SNP associations with ventricular dysfunction after primary coronary artery bypass graft surgery.** Genomic inflation factor lambdas derived from these Q-Q plots were used to adjust GWAS association findings for potential population stratification: allelic model lambda = 1.08, additive model (trend test) lambda = 1.07, dominant model lambda = 1.05, and recessive model lambda = 1.00.
(TIF)

Figure S2 **Examples of single nucleotide polymorphism (SNP) genotyping cluster plots with good and poor differentiation between heterozygotes and homozygotes.** SNP = single nucleotide polymorphism; NC = no genotype call; AA = homozygote genotype for one allele; AB = heterozygote genotype call; BB homozygote genotype call for other allele Two investigators visually inspected intensity cluster plots for genotyping calls for those SNPs with GC adjusted association P values<1.0×10^−4^ in the GWAS (allelic, additive and dominant model). Intensity cluster plots for recessive model SNPs with association GC adjusted P values<10^−5^ in the GWAS as determined by chi-square tests were also reviewed, and P values for SNPs with good cluster plot differentiation were then assessed using Fisher's exact tests (SNPs with P<10^−4^ by Fisher's Exact tests; Table S1). Cluster plots were categorized as having good or poor differentiation between homozygote and heterozygote calls (plots were derived from GWAS Affymetrix 6.0 Genome-Wide Human SNP Array case and control data called together using Birdseed version 2 calling algorithm). SNPs with poorly differentiated intensity cluster plots were dropped from further consideration in validation and replication studies (33 SNPs for allelic model, 35 SNPs additive model, 24 SNPs for recessive model). The X axis represents contrast ([A−B]/[A+B]), and the Y axis represents strength (log[A+B]), where A and B are the summarized intensities of the two alleles for one sample. Summarized intensities means a single value that summarizes the intensities of all the many oligonucleotide probes on the chip that contribute to measuring this binary allele at this SNP locus.
(TIF)

Information S1 **Supplementary Methods.**
(DOC)

Table S1 **Genome wide association study results: SNP associations with ventricular dysfunction after primary coronary artery bypass graft surgery.**
(DOC)

Table S2 **Validation study results: SNP associations with ventricular dysfunction after primary coronary artery bypass graft surgery in 786 European ancestry men from the CABG Genomics Study Cohort.** 63 SNPs were selected from 34 genetic loci identified to be associated with ventricular dysfunction in the GWAS.
(PDF)

Table S3 **Vanderbilt cohort replication study results: 17 SNP associations (13 genetic loci) with ventricular dysfunction after primary coronary artery bypass graft surgery in 337 European ancestry men.**
(DOC)

Table S4 **CABG Genomics cohort replication study results: 19 SNP associations (15 genetic loci) with ventricular dysfunction after primary coronary artery bypass graft surgery in 980 European ancestry men.**
(DOC)

Table S5 **Covariate adjusted meta-analysis results: 17 SNP associations (13 genetic loci) with ventricular dysfunction after primary coronary artery bypass graft surgery in 1388 European ancestry men.**
(DOC)
