# Supplementary figures and images for: Genome-Wide Assessment for Genetic Variants Associated with Ventricular Dysfunction after Primary Coronary Artery Bypass Graft Surgery
Source: PLoS One. 2011 Sep 30;6(9):e24593. doi: 10.1371/journal.pone.0024593 (PMC3184087; doi:10.1371/journal.pone.0024593)

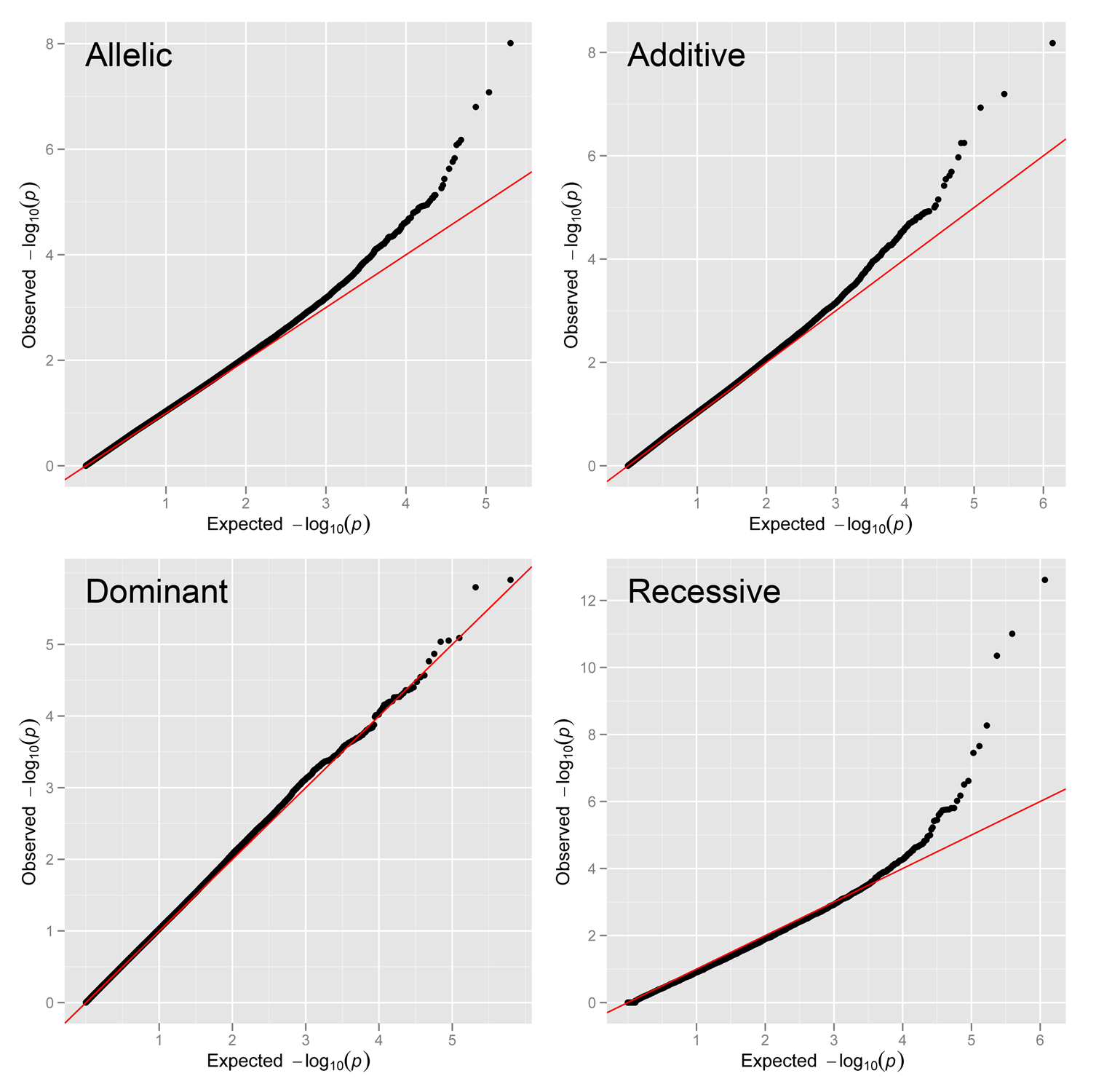

Supplement: Figure S1 — Genome wide association study Q-Q plots of the expected and observed –log P values for SNP associations with ventricular dysfunction after primary coronary artery bypass graft surgery. Genomic inflation factor lambdas derived from these Q-Q plots were used to adjust GWAS association findings for potential population stratification: allelic model lambda = 1.08, additive model (trend test) lambda = 1.07, dominant model lambda = 1.05, and recessive model lambda = 1.00. (TIF) [file pone.0024593.s001.tif]

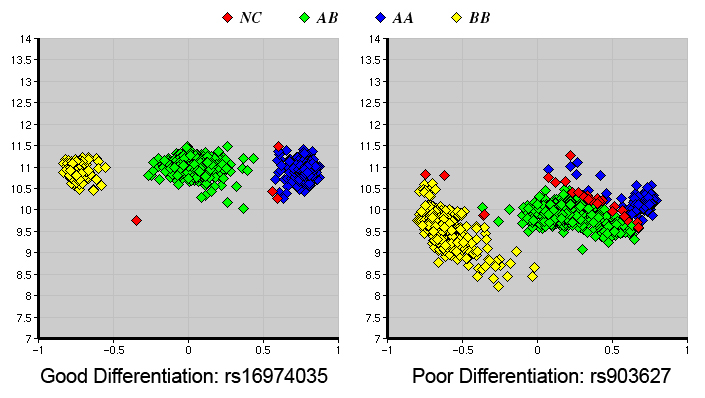

Supplement: Figure S2 — Examples of single nucleotide polymorphism (SNP) genotyping cluster plots with good and poor differentiation between heterozygotes and homozygotes. SNP = single nucleotide polymorphism; NC = no genotype call; AA = homozygote genotype for one allele; AB = heterozygote genotype call; BB homozygote genotype call for other allele Two investigators visually inspected intensity cluster plots for genotyping calls for those SNPs with GC adjusted association P values<1.0×10^−4^ in the GWAS (allelic, additive and dominant model). Intensity cluster plots for recessive model SNPs with association GC adjusted P values<10−5 in the GWAS as determined by chi-square tests were also reviewed, and P values for SNPs with good cluster plot differentiation were then assessed using Fisher's exact tests (SNPs with P<10−4 by Fisher's Exact tests; Table S1). Cluster plots were categorized as having good or poor differentiation between homozygote and heterozygote calls (plots were derived from GWAS Affymetrix 6.0 Genome-Wide Human SNP Array case and control data called together using Birdseed version 2 calling algorithm). SNPs with poorly differentiated intensity cluster plots were dropped from further consideration in validation and replication studies (33 SNPs for allelic model, 35 SNPs additive model, 24 SNPs for recessive model). The X axis represents contrast ([A−B]/[A+B]), and the Y axis represents strength (log[A+B]), where A and B are the summarized intensities of the two alleles for one sample. Summarized intensities means a single value that summarizes the intensities of all the many oligonucleotide probes on the chip that contribute to measuring this binary allele at this SNP locus. (TIF) [file pone.0024593.s002.tif]
