# Supplementary material for: Genome-Wide Assessment for Genetic Variants Associated with Ventricular Dysfunction after Primary Coronary Artery Bypass Graft Surgery
Source: PLoS One. 2011 Sep 30;6(9):e24593. doi: 10.1371/journal.pone.0024593 (PMC3184087; doi:10.1371/journal.pone.0024593)
Supplement: Information S1 — Supplementary Methods. (DOC) [file pone.0024593.s003.doc]

**Supporting Information S1:**

Supplementary Methods: Genome-wide assessment for genetic variants associated with ventricular dysfunction after primary coronary artery bypass graft surgery

**Descriptions of the Cohorts Providing the Gene Association Study Data**

The CABG Genomics Program enrolled 1922 patients aged 21-90 years undergoing primary CABG surgery without planned concurrent valve surgery between August 2001 and January 2009 ([*http://clinicaltrials.gov/show/NCT00281164*](http://clinicaltrials.gov/show/NCT00281164)). The Vanderbilt Cardiac Surgery Registry (VCSR) enrolled 1279 patients older than 18 years undergoing CABG, valve, ascending aorta, or congenital heart surgery at Vanderbilt University Medical Center from November 1999 to November 2004. The multiple sclerosis study cohort provided de-identified Affymetrix 6.0 Genome-Wide Human SNP Array control data (Affymetrix, Santa Clara, CA) for 1298 enrolled subjects of European ancestry (1123 control subjects were analyzed after genotyping quality control: 28% male; 78% with multiple sclerosis).

**Genome-Wide Association Study Subject Exclusions to Control for Population Structure**

In addition to excluding subjects who were not of Northern or Southern European ancestry, standard PLINK methods were used to exclude subjects who were population stratification outliers or appeared to have cryptic relatedness. Population stratification outliers with excessive genome-wide identity-by-state (IBS) distance to their nearest neighbors were identified using the PLINK command --cluster --neighbor. Pairs of subjects with an excessive proportion of genome-wide identity-by-descent (IBD) sharing were estimated from the given IBS information using the PLINK command --genome.

**Meta-analysis of Subjects in the Validation and Replication Studies**

The 17 SNPs (13 genetic loci) genotyped in both the CABG Genomics and the Vanderbilt replication studies were assessed for association with VnD in a meta-analysis first of the two replication studies, and then of all VnD cases and controls included in the CABG Genomics validation study, the CABG Genomics replication study, and the Vanderbilt replication study (PLINK version 1.07).[1] The meta-analyses were conducted using the random effects model of covariate adjusted ORs. Cochran’s Q statistic and the heterogeneity index (I^2^) were used to assess heterogeneity of SNP associations between replication studies (CABG Genomics and Vanderbilt) and then across institutions for the meta-analysis of subjects genotyped for the CABG Genomics validation study and the CABG Genomics and Vanderbilt replication studies. There were 818 CABG Genomics BWH subjects, 233 CABG Genomics THI subjects, and 337 Vanderbilt subjects for the meta-analysis of the validation and replication study data.

**Reference**

1. Purcell S, Neale B, Todd-Brown K, Thomas L, Ferreira MA, et al. (2007) PLINK: a tool set for whole-genome association and population-based linkage analyses. Am J Hum Genet 81: 559-575.
