# Supplementary material for: Genome-Wide Assessment for Genetic Variants Associated with Ventricular Dysfunction after Primary Coronary Artery Bypass Graft Surgery
Source: PLoS One. 2011 Sep 30;6(9):e24593. doi: 10.1371/journal.pone.0024593 (PMC3184087; doi:10.1371/journal.pone.0024593)
Supplement: Table S1 — Genome wide association study results: SNP associations with ventricular dysfunction after primary coronary artery bypass graft surgery. (DOC) [file pone.0024593.s004.doc]

**Supporting Information Table S1. *Genome wide association study results: SNP associations with ventricular dysfunction after primary coronary artery bypass graft surgery.***

| SNP | Chromosome #, Location NCBI hg36 | Gene | MAF VnD Cases (n=76) | MAF Controls (n=1123) | Allelic Model GC adjusted P Value | Additive Model GC Adjusted P value | Dominant Model GC Adjusted P value | Recessive Model Fisher’s Exact P value |
| --- | --- | --- | --- | --- | --- | --- | --- | --- |
| **SNPs P < 10^-4^ for Allelic Model** | | | | | | | | |
| **rs7549755** | Chr1:91930396 | *TGFBR3* | 0.33 | 0.19 | 9.95x10^-5^ | 5.60x10^-5^ | 6.50x10^-5^ |  |
| rs3754021 | Chr1:91931339 | *TGFBR3* | 0.31 | 0.18 | 8.65x10^-5^ | 5.60x10^-5^ | 9.82x10^-5^ |  |
| **rs2798601** | Chr1:153046309 | *KCNN3* | 0.29 | 0.16 | 7.96x10^-5^ | 7.10x10^-5^ |  |  |
| rs6674725 | Chr1:175465441 | *FAM5B* | 0.11 | 0.26 | 6.67x10^-5^ | 6.28x10^-5^ |  |  |
| **rs12744134** | Chr1:175490296 | *FAM5B* | 0.11 | 0.25 | 8.85x10^-5^ | 8.24x10^-5^ |  |  |
| **rs10752878** | Chr1:180848998 | *RGS16* | 0.27 | 0.13 | 3.62x10^-6^ | 4.54x10^-6^ |  |  |
| **rs1287820** | Chr1:180855954 |  | 0.34 | 0.20 | 5.08x10^-5^ | 4.69x10^-5^ |  |  |
| rs11589747 | Chr1:183982017 | *HMCN1* | 0.17 | 0.07 | 1.21x10^-5^ | 1.44x10^-5^ |  |  |
| rs1994391 | Chr2:77843073 |  | 0.08 | 0.02 | 3.10x10^-5^ | 3.12x10^-5^ |  |  |
| rs1017931 | Chr2:77856791 |  | 0.09 | 0.03 | 4.09x10^-5^ | 3.83x10^-5^ |  |  |
| rs2171325 | Chr2:77879084 |  | 0.09 | 0.02 | 8.39x10^-6^ | 8.09x10^-6^ |  |  |
| rs16829861 | Chr2:134446170 |  | 0.28 | 0.15 | 2.61x10^-5^ | 2.35x10^-5^ |  |  |
| rs41321847 | Chr3:34902008 |  | 0.23 | 0.11 | 2.42x10^-5^ | 2.86 x10^-5^ |  |  |
| rs11129625 | Chr3:34914897 |  | 0.23 | 0.11 | 2.75x10^-5^ | 3.43x10^-5^ |  |  |
| rs885314 | Chr3:34915736 |  | 0.23 | 0.11 | 2.55x10^-5^ | 2.99x10^-5^ |  |  |
| **rs17691914** | Chr3:34937807 |  | 0.21 | 0.07 | 3.42x10^-8^ | 2.14x10^-8^ |  |  |
| **rs9835451** | Chr3:34946568 |  | 0.23 | 0.11 | 5.55x10^-6^ | 5.33x10^-6^ |  |  |
| rs11129626 | Chr3:34950049 |  | 0.23 | 0.11 | 1.81x10^-5^ | 2.36x10^-5^ |  |  |
| rs4678726 | Chr3:34951024 |  | 0.23 | 0.11 | 1.63x10^-5^ | 2.00x10^-5^ |  |  |
| rs1979406 | Chr3:35014326 |  | 0.21 | 0.08 | 4.55x10^-7^ | 3.13x10^-7^ |  |  |
| rs17032625 | Chr3:35052013 |  | 0.21 | 0.08 | 2.50x10^-7^ | 1.78x10^-7^ |  |  |
| **rs17358517** | Chr3:59660772 |  | 0.38 | 0.22 | 2.04x10^-5^ | 2.67x10^-5^ | 8.38x10^-5^ |  |
| rs17292696 | Chr3:59661326 |  | 0.38 | 0.22 | 2.23x10^-5^ | 3.08x10^-5^ | 8.30x10^-5^ |  |
| rs11924953 | Chr3:59661465 |  | 0.38 | 0.22 | 3.21x10^-5^ | 4.13x10^-5^ |  |  |
| rs4420916 | Chr3:75072673 |  | 0.34 | 0.20 | 7.11x10^-5^ | 6.64x10^-5^ |  |  |
| **rs9863767** | Chr3:75164647 |  | 0.34 | 0.20 | 4.19x10^-5^ | 3.95x10^-5^ |  |  |
| rs4635750 | Chr3:75180559 |  | 0.34 | 0.20 | 4.63x10^-5^ | 4.71x10^-5^ |  |  |
| rs12486094 | Chr3:75210242 |  | 0.34 | 0.20 | 8.11x10^-5^ | 8.93x10^-5^ |  |  |
| **rs9837024** | Chr3:78534327 |  | 0.36 | 0.21 | 2.52x10^-5^ | 2.44x10^-5^ |  |  |
| rs13322524 | Chr3:78578410 |  | 0.37 | 0.21 | 2.98x10^-5^ | 3.01x10^-5^ |  |  |
| rs16844402 | Chr3:103105950 |  | 0.11 | 0.04 | 2.64x10^-5^ | 2.40x10^-5^ |  |  |
| **rs500910** | Chr3:184360726 | *LAMP3* | 0.20 | 0.37 | 4.01x10^-5^ | 2.47x10^-5^ |  |  |
| rs675924 | Chr3:184342095 | *LAMP3* | 0.20 | 0.34 |  |  | 6.76x10^-5^ |  |
| rs1464574 | Chr3:184361532 | *LAMP3* | 0.32 | 0.48 |  |  | 2.88x10^-6^ |  |
| **rs1965484** | Chr3:184361591 | *LAMP3* | 0.32 | 0.48 |  | 9.71x10^-5^ | 2.30x10^-6^ |  |
| rs55918414 | Chr4:111508473 |  | 0.11 | 0.04 | 8.45x10^-5^ | 9.40x10^-5^ |  |  |
| rs12513172 | Chr4:111519959 |  | 0.11 | 0.04 | 9.71x10^-5^ |  |  |  |
| **rs42430*** | Chr5:80478480 | *RASGRF2* | 0.33 | 0.19 | 4.72x10^-5^ | 3.52x10^-5^ | 2.21x10^-5^ |  |
| rs9285916 | Chr5:129432844 |  | 0.25 | 0.13 | 2.48x10^-5^ | 2.98x10^-5^ |  |  |
| **rs4836493** | Chr5:129499621 | *CSS3* | 0.13 | 0.04 | 4.18x10^-6^ | 6.19x10^-6^ |  |  |
| rs2546316 | Chr5:163278278 |  | 0.47 | 0.30 | 2.68x10^-5^ | 2.58x10^-5^ |  | 3.96x10^-5^ |
| **rs1017213** | Chr5:163281156 |  | 0.46 | 0.29 | 3.31x10^-5^ | 2.65x10^-5^ |  |  |
| rs2544995 | Chr5:163281659 |  | 0.45 | 0.29 | 5.66x10^-5^ | 4.34x10^-5^ |  |  |
| rs1895178 | Chr5:163283841 |  | 0.45 | 0.29 | 5.07x10^-5^ | 3.73x10^-5^ |  |  |
| rs1012958 | Chr6:85179681 |  | 0.48 | 0.32 | 6.10x10^-5^ | 7.83x10^-5^ |  |  |
| **rs6459959** | Chr7:155390912 |  | 0.47 | 0.33 | 1.73x10^-6^ | 1.36x10^-6^ |  | 2.24x10^-5^ |
| **rs6459961** | Chr7:155391016 |  | 0.49 | 0.32 | 2.11x10^-6^ | 2.49x10^-6^ |  | 4.56x10^-5^ |
| rs11785994 | Chr8:13920420 |  | 0.09 | 0.02 | 1.08x10^-5^ | 1.85x10^-5^ |  |  |
| **rs1044714*** | Chr8:124334108 |  | 0.37 | 0.22 | 9.10x10^-5^ | 7.71x10^-5^ |  | 1.40x10^-5^ |
| rs13248086 | Chr8:124448936 | *ATAD2* | 0.36 | 0.22 |  |  |  | 6.54x10^-5^ |
| rs13291796 | Chr9:81354825 |  | 0.26 | 0.14 | 4.71x10^-5^ | 4.83x10^-5^ |  |  |
| rs3849130 | Chr9:114258209 | *HSDL2* | 0.12 | 0.04 | 9.04x10^-5^ | 1.00x10^-4^ |  |  |
| rs10759566 | Chr9:114258962 | *HSDL2* | 0.12 | 0.04 | 8.77x10^-5^ | 9.71x10^-5^ |  |  |
| rs7846977 | Chr9:114259888 | *HSDL2* | 0.12 | 0.04 | 8.77x10^-5^ | 9.71x10^-5^ |  |  |
| rs7032579 | Chr9:114263119 | *HSDL2* | 0.12 | 0.04 | 8.77x10^-5^ | 9.71x10^-5^ |  |  |
| rs2900543 | Chr9:114264220 | *HSDL2* | 0.12 | 0.04 | 8.77x10^-5^ | 9.71x10^-5^ |  |  |
| rs10981416 | Chr9:114268276 | *HSDL2* | 0.12 | 0.04 | 8.77x10^-5^ | 9.71x10^-5^ |  |  |
| rs10283468 | Chr9:114283504 | *HSDL2* | 0.12 | 0.04 | 8.77x10^-5^ | 9.71x10^-5^ |  |  |
| rs7023765 | Chr9:114284243 | *HSDL2* | 0.12 | 0.04 | 8.77x10^-5^ | 9.71x10^-5^ |  |  |
| **rs6477941** | Chr9:114292170 | *KIAA1958* | 0.12 | 0.04 | 5.37x10^-5^ | 5.26x10^-5^ |  |  |
| rs3849131 | Chr9:114298531 | *KIAA1958* | 0.12 | 0.04 | 7.43x10^-5^ | 8.30x10^-5^ |  |  |
| rs10739360 | Chr9:114312813 | *KIAA1958* | 0.12 | 0.04 | 8.77x10^-5^ | 9.71x10^-5^ |  |  |
| rs17436666 | Chr10:17421196 | *ST8SIA6* | 0.07 | 0.02 | 7.05x10^-5^ | 7.91x10^-5^ |  |  |
| rs41349746 | Chr12:23660749 |  | 0.08 | 0.02 | 7.05x10^-5^ | 6.92 x10^-5^ |  |  |
| **rs7975290** | Chr12:26505185 | *ITPR2* | 0.13 | 0.05 | 8.41x10^-5^ | 6.19x10^-5^ |  |  |
| rs1993997 | Chr12:64680084 |  | 0.42 | 0.27 |  | 9.62x10^-5^ |  |  |
| rs922628 | Chr12:64681102 |  | 0.42 | 0.26 | 4.20x10^-5^ | 3.61x10^-5^ | 9.40x10^-5^ |  |
| **rs10773689** | Chr12:128686079 | *TMEM132D* | 0.23 | 0.41 | 2.40x10^-5^ | 3.62 x10^-5^ |  |  |
| rs10773690 | Chr12:128686175 | *TMEM132D* | 0.23 | 0.41 | 1.62x10^-5^ | 2.44 x10^-5^ |  |  |
| rs12297578 | Chr12:128690377 | *TMEM132D* | 0.34 | 0.47 | 1.82x10^-5^ | 3.90x10^-5^ | 7.36x10^-5^ |  |
| **rs11060480** | Chr12:128692468 | *TMEM132D* | 0.34 | 0.48 | 4.15x10^-5^ | 7.24x10^-5^ |  |  |
| rs585618 | Chr12:128711360 | *TMEM132D* | 0.35 | 0.47 | 3.34x10^-5^ | 6.73x10^-5^ |  |  |
| rs605142 | Chr12:128711711 | *TMEM132D* | 0.35 | 0.47 | 2.49x10^-5^ | 4.49x10^-5^ |  |  |
| rs196130 | Chr13:108250081 | *MYR8* | 0.20 | 0.10 | 7.05x10^-5^ | 5.68x10^-5^ |  |  |
| **rs10519861** | Chr15:31773350 | *RYR3* | 0.44 | 0.39 | 6.52x10^-5^ | 7.35x10^-5^ |  |  |
| **rs8027394** | Chr15:76404968 |  | 0.47 | 0.31 | 6.71x10^-5^ | 4.43x10^-5^ |  | 6.73 x10^-5^ |
| **rs7183919*** | Chr15:84981864 |  | 0.22 | 0.11 | 4.79x10^-5^ | 6.81x10^-5^ |  |  |
| rs4984505 | Chr15:94492655 |  | 0.24 | 0.41 | 6.55x10^-5^ | 9.02 x10^-5^ |  |  |
| rs17136692 | Chr16:4008814 | *ADCY9* | 0.18 | 0.08 | 9.57x10^-5^ |  |  |  |
| **rs8058644** | Chr16:48454181 |  | 0.16 | 0.06 | 1.95x10^-6^ | 1.37 x10^-6^ |  |  |
| rs2302299 | Chr17:3672648 | *HSA277841* | 0.36 | 0.22 | 7.78x10^-5^ | 7.43x10^-5^ |  |  |
| **rs16974035** | Chr18:10269356 |  | 0.48 | 0.31 | 5.00x10^-5^ | 3.48x10^-5^ |  |  |
| **rs2062980** | Chr18:60573886 |  | 0.45 | 0.38 | 8.49x10^-5^ | 9.10x10^-5^ |  |  |
| rs34985812 | Chr20:60769970 | *SLCO4A1* | 0.14 | 0.05 | 5.58x10^-5^ | 5.24x10^-5^ |  |  |
| rs2018682 | Chr22:19647432 | *AIFL* | 0.46 | 0.37 | 5.00x10^-5^ | 5.79x10^-5^ |  |  |
| **SNPs P < 10^-4^ for only Additive Model** | | | | | | | | |
| rs4713212 | Chr6:11269394 |  | 0.43 | 0.28 |  | 9.25x10^-5^ |  |  |
| rs2617841 | Chr16:54930723 | *GNAO1* | 0.13 | 0.28 |  | 8.95x10^-5^ |  |  |
| **SNPs P < 10^-4^ for only Dominant Model** | | | | | | | | |
| rs10911717 | Chr1:183635854 |  | 0.29 | 0.43 |  |  | 7.85x10^-5^ |  |
| rs7545878 | Chr1:183593483 |  | 0.26 | 0.42 |  |  | 9.62x10^-5^ |  |
| rs7512337 | Chr1:183602155 |  | 0.26 | 0.42 |  |  | 9.59x10^-5^ |  |
| rs10911722 | Chr1:183648214 |  | 0.28 | 0.43 |  |  | 8.26x10^-5^ |  |
| rs6705249 | Chr2:117238325 |  | 0.28 | 0.43 |  |  | 6.75x10^-5^ |  |
| **rs6766797** | Chr3:67266318 |  | 0.24 | 0.41 |  |  | 1.36x10^-5^ |  |
| rs755791 | Chr11:116938896 | *DSCAML1* | 0.49 | 0.36 |  |  | 1.53x10^-5^ |  |
| rs2010473 | Chr11:116938949 | *DSCAML1* | 0.49 | 0.34 |  |  | 1.47x10^-5^ |  |
| rs364465 | Chr17:27043392 |  | 0.41 | 0.28 |  |  | 6.18x10^-5^ |  |
| rs8069504 | Chr17:30798378 | *FLJ31952* | 0.35 | 0.50 |  |  | 2.78x10^-5^ |  |
| rs868929 | Chr17:69484870 |  | 0.28 | 0.41 |  |  | 8.39x10^-5^ |  |
| rs8081998 | Chr17:69486206 |  | 0.28 | 0.42 |  |  | 4.52x10^-5^ |  |
| **rs6567111*** | Chr18:55474770 | *CCBE1* | 0.24 | 0.41 |  |  | 4.28x10^-5^ |  |
| rs1652065 | Chr18:55494646 | *CCBE1* | 0.23 | 0.38 |  |  | 5.22x10^-5^ |  |
| **SNPs P < 10^-4^ for only Recessive Model** | | | | | | | | |
| **rs7430827** | Chr3:6617993 |  | 0.42 | 0.34 |  |  |  | 5.19x10^-5^ |
| rs7637316 | Chr3:6618607 |  | 0.43 | 0.34 |  |  |  | 2.36x10^-5^ |
| **rs9831754** | Chr3:78436281 |  | 0.30 | 0.18 |  |  |  | 1.12x10^-5^ |
| rs7429509 | Chr3:152583857 | *P2RY12* | 0.26 | 0.19 |  |  |  | 5.96x10^-5^ |
| **rs17597256** | Chr3:198252251 | *DLG1* | 0.47 | 0.35 |  |  |  | 3.64x10^-5^ |
| **rs4242051** | Chr5:54234532 |  | 0.38 | 0.26 |  |  |  | 7.28x10^-5^ |
| rs10256183 | Chr7:52189816 |  | 0.49 | 0.36 |  |  |  | 2.14x10^-5^ |
| rs7820759 | Chr8:40759570 | *ZMAT4* | 0.38 | 0.25 |  |  |  | 9.04x10^-5^ |
| **rs10104640** | Chr8:40762563 | *ZMAT4* | 0.37 | 0.25 |  |  |  | 8.07x10^-5^ |
| **rs10857508** | Chr10:50443270 |  | 0.16 | 0.09 |  |  |  | 3.64x10^-5^ |
| rs11527800 | Chr10:50464443 |  | 0.16 | 0.09 |  |  |  | 2.27x10^-5^ |
| rs11101175 | Chr10:50468566 |  | 0.16 | 0.09 |  |  |  | 5.66x10^-5^ |
| rs11023934 | Chr11:16382534 | *SOX6* | 0.29 | 0.20 |  |  |  | 9.12x10^-5^ |
| **rs10832607** | Chr11:16419343 | *SOX6* | 0.29 | 0.20 |  |  |  | 8.51x10^-5^ |
| **rs10500830** | Chr11:16441827 | *SOX6* | 0.29 | 0.20 |  |  |  | 2.34x10^-4^ |
| rs4132991 | Chr11:16524782 |  | 0.26 | 0.19 |  |  |  | 8.85x10^-5^ |
| **rs12279572** | Chr11:117458170 | *TMPRSS4* | 0.36 | 0.28 |  |  |  | 1.92x10^-5^ |
| rs9317632 | Chr13:66441449 | *PCDH9* | 0.26 | 0.16 |  |  |  | 4.99x10^-5^ |
| rs12147730 | Chr14:81989111 |  | 0.42 | 0.32 |  |  |  | 2.01x10^-5^ |
| rs10145849 | Chr14:82011744 |  | 0.47 | 0.37 |  |  |  | 6.15x10^-5^ |
| rs10146004 | Chr14:82012141 |  | 0.44 | 0.34 |  |  |  | 2.88x10^-5^ |
| **rs2016367** | Chr20:50276712 |  | 0.29 | 0.20 |  |  |  | 7.08x10^-5^ |

GC adjusted = genomic control (lambda) adjusted; MAF = minor allele frequency; SNP = single nucleotide polymorphism; VnD = ventricular dysfunction;

**Bold font** signifies SNPs assessed in CABG Genomics validation study (Supporting Information Table S3) unless noted by *.

Alternate shading highlights SNPs within different genetic loci.

* Signifies SNPs that could not be genotyped using Sequenom genotyping platform for CABG Genomics validation study, but alternate SNPs in strong linkage dysequilibium (r^2^>0.80) with the GWAS SNP were selected for genotyping in the CABG Genomics validation study (Supporting Information Table S3).
