# Supplementary material for: Genome-Wide Assessment for Genetic Variants Associated with Ventricular Dysfunction after Primary Coronary Artery Bypass Graft Surgery
Source: PLoS One. 2011 Sep 30;6(9):e24593. doi: 10.1371/journal.pone.0024593 (PMC3184087; doi:10.1371/journal.pone.0024593)
Supplement: Table S2 — Validation study results: SNP associations with ventricular dysfunction after primary coronary artery bypass graft surgery in 786 European ancestry men from the CABG Genomics Study Cohort. 63 SNPs were selected from 34 genetic loci identified to be associated with ventricular dysfunction in the GWAS. (PDF) [file pone.0024593.s005.pdf]

Supporting Information Table S2. ***Validation study results: SNP associations with ventricular dysfunction after primary coronary artery bypass graft surgery in 786 European ancestry men from the CABG Genomics Study Cohort.*** *(63 SNPs were selected from 34 genetic loci identified to be associated with ventricular dysfunction in the GWAS)*

**SNP**	**Chr #, Location** NCBI hg36	**Gene**	**GWAS or Additional SNP**	**Minor Allele/ Major Allele**	**MAF VnD Cases** (n=71)***/ Controls** (n=715)	**Genetic Model**	**Unadjusted Odds Ratio**	**Univariate Asymptotic P value**	**Univariate Permuted Point-wise P value**	**MV Adjusted Odds Ratio**	**MV Adjusted Asymptotic P value**	**MV Adjusted Point-wise P value**
rs7549755	Chr 1,	*TGFBR3*	GWAS	C/T	0.32/0.21	Allelic	1.78	0.002	0.003			
	91,930,396					Additive	1.81	0.002	0.003	1.65	0.02	0.02
					GWAS	Dominant	2.23	0.002	0.002	2.14	0.005	0.005
					0.33/0.19	Recessive	1.67	0.30	0.31	1.16	0.78	0.75
rs2765888	Chr 1,		Additional	T/C	0.08/0.11	Allelic	0.68	0.24	0.26			
	91,934,880					Additive	0.68	0.24	0.26	0.84	0.61	0.61
						Dominant	0.63	0.19	0.17	0.77	0.48	0.50
						Recessive	1.25	0.83	0.37	1.96	0.54	0.20
rs6690388	Chr 1,	*KCNN3*	Additional	C/T	0.30/0.20	Allelic	1.78	0.003	0.002			
	153,041,071					Additive	1.75	0.004	0.003	1.70	0.01	0.008
						Dominant	2.16	0.002	0.001	2.24	0.003	0.002
						Recessive	1.62	0.33	0.31	1.20	0.74	0.62
rs6426944	Chr 1,	*KCNN3*	Additional	A/G	0.37/0.25	Allelic	1.76	0.002	0.002			
	153,043,036					Additive	1.77	0.002	0.002	1.81	0.005	0.006
						Dominant	2.35	0.001	0.001	2.12	0.007	0.007
						Recessive	1.56	0.30	0.26	2.00	0.15	0.12
rs1702176	Chr 1,	*KCNN3*	Additional	T/C	0.25/0.27	Allelic	0.94	0.74	0.78			
	153,043,781					Additive	0.94	0.74	0.71	0.83	0.41	0.41
						Dominant	1.03	0.90	0.98	0.87	0.61	0.62
						Recessive	0.54	0.31	0.23	0.53	0.32	0.33
rs2798601	Chr 1,	*KCNN3*	GWAS	A/G	0.30/0.18	Allelic	1.94	0.0006	0.001			
	153,046,309					Additive	1.92	0.0009	0.001	1.95	0.003	0.002
					GWAS	Dominant	2.16	0.002	0.002	2.11	0.006	0.004
					0.29/0.16	Recessive	2.66	0.04	0.04	2.92	0.05	0.04
rs1013219	Chr 1,	*FAM5B*	Additional	A/G	0.36/0.40	Allelic	0.85	0.38	0.43			
	175,479,499					Additive	0.85	0.38	0.42	0.81	0.28	0.27
						Dominant	1.10	0.72	0.73	1.01	0.97	1.00
						Recessive	0.38	0.04	0.03	0.36	0.04	0.04
rs12744134	Chr 1,	*FAM5B*	GWAS	A/G	0.11/0.23	Allelic	0.42	0.001	0.001			
	175,490,296					Additive	0.42	0.002	0.0008	0.39	0.002	0.001
					GWAS	Dominant	0.43	0.004	0.003	0.39	0.003	0.002
					0.11/0.25	Recessive	-	1.00	0.98	-	1.00	0.98
rs10752878	Chr 1,	*RGS16*	GWAS	G/T	0.25/0.14	Allelic	2.09	0.0003	0.0006			
	180,848,998					Additive	1.98	0.0007	0.0008	2.10	0.001	0.0008
					GWAS	Dominant	2.16	0.003	0.002	2.29	0.003	0.004
					0.27/0.13	Recessive	3.38	0.01	0.01	3.86	0.02	0.01

**rs1287820**	Chr 1,<br>180,855,954		GWAS	G/C	0.32/0.20<br><br>GWAS<br>0.34/0.20	Allelic<br>Additive<br>Dominant<br>Recessive	1.96<br>2.00<br>2.19<br>3.02	0.0004<br>0.0004<br>0.002<br>0.01	0.001<br>0.0004<br>0.001<br>0.02	 2.26<br>2.53<br>3.70	 0.0002<br>0.0008<br>0.008	 0.0004<br>0.0002<br>0.008
rs7646796	Chr 3,<br>6,615,547		Additional	T/C	0.45/0.43	Allelic<br>Additive<br>Dominant<br>Recessive	1.08<br>1.07<br>0.79<br>1.66	0.68<br>0.69<br>0.36<br>0.07	0.72<br>0.71<br>0.36<br>0.07	 1.00<br>0.74<br>1.45	 0.99<br>0.28<br>0.23	 0.99<br>0.29<br>0.24
rs7430827	Chr 3,<br>6,617,993		GWAS	C/A	0.42/0.36<br><br>GWAS<br>0.42/0.34	Allelic<br>Additive<br>Dominant<br>Recessive	1.29<br>1.28<br>0.86<br>2.76	0.16<br>0.17<br>0.53<br>0.0004	0.17<br>0.16<br>0.62<br>0.001	 1.45<br>1.08<br>3.03	 0.05<br>0.77<br>0.0005	 0.05<br>0.78<br>0.0002
**rs17691914**	Chr 3,<br>34,937,807		GWAS	G/A	0.19/0.08<br><br>GWAS<br>0.21/0.07	Allelic<br>Additive<br>Dominant<br>Recessive	2.74<br>2.74<br>3.53<br>-	$9.3X10^{-6}$<br>$2.7X10^{-5}$<br>$2.2X10^{-6}$<br>1.00	<0.0001<br>0.0002<br>0.0002<br>0.60	 2.96<br>3.76<br>-	 $6.0X10^{-5}$<br>$1.1X10^{-5}$<br>1.00	 0.0002<br>0.0002<br>0.98
**rs9835451**	Chr 3,<br>34,946,568		GWAS	G/A	0.22/0.11<br><br>GWAS<br>0.23/0.11	Allelic<br>Additive<br>Dominant<br>Recessive	2.17<br>2.14<br>2.76<br>0.77	0.0003<br>0.0005<br>$8.1X10^{-5}$<br>0.80	0.0005<br>0.0006<br>0.0002<br>0.44	 2.29<br>2.62<br>1.75	 0.001<br>0.0007<br>0.60	 0.0008<br>0.0008<br>0.33
**rs17358517**	Chr 3,<br>59,660,772		GWAS	T/C	0.38/0.23<br><br>GWAS<br>0.38/0.22	Allelic<br>Additive<br>Dominant<br>Recessive	2.07<br>2.07<br>2.57<br>2.52	$5.8X10^{-5}$<br>$9.6X10^{-5}$<br>0.0003<br>0.02	0.0005<br>0.0002<br>0.0002<br>0.009	 2.14<br>2.72<br>2.61	 0.0002<br>0.0004<br>0.02	 0.0004<br>0.0004<br>0.01
**rs17061085**	Chr 3,<br>59,666,765		Additional	A/G	0.26/0.13	Allelic<br>Additive<br>Dominant<br>Recessive	2.42<br>2.04<br>2.69<br>2.59	$1.2X10^{-5}$<br>0.0001<br>0.0001<br>0.03	0.0003<br>0.0004<br>0.0004<br>0.03	 2.07<br>2.76<br>2.61	 0.0004<br>0.0003<br>0.05	 0.0004<br>0.0002<br>0.04
rs6774674	Chr 3,<br>67,263,367		Additional	C/T	0.32/0.39	Allelic<br>Additive<br>Dominant<br>Recessive	0.71<br>0.71<br>0.62<br>0.72	0.07<br>0.07<br>0.05<br>0.39	0.07<br>0.07<br>0.05<br>0.42	 0.67<br>0.56<br>0.73	 0.06<br>0.03<br>0.44	 0.05<br>0.04<br>0.43
rs6766797	Chr 3,<br>67,266,318		GWAS	A/T	0.27/0.36<br><br>GWAS<br>0.24/0.41	Allelic<br>Additive<br>Dominant<br>Recessive	0.64<br>0.63<br>0.51<br>0.75	0.02<br>0.02<br>0.007<br>0.49	0.02<br>0.02<br>0.01<br>0.51	 0.60<br>0.46<br>0.82	 0.02<br>0.004<br>0.66	 0.02<br>0.005<br>0.70
rs9863767	Chr 3,<br>75,164,647		GWAS	A/C	0.33/0.22<br><br>GWAS<br>0.34/0.20	Allelic<br>Additive<br>Dominant<br>Recessive	1.72<br>1.79<br>1.88<br>2.68	0.004<br>0.003<br>0.01<br>0.03	0.004<br>0.004<br>0.01<br>0.02	 1.86<br>1.92<br>3.06	 0.004<br>0.02<br>0.02	 0.004<br>0.01<br>0.02
rs9831754	Chr 3,<br>78,436,281		GWAS	G/T	0.29/0.20<br><br>GWAS<br>0.30/0.18	Allelic<br>Additive<br>Dominant<br>Recessive	1.65<br>1.67<br>1.53<br>3.82	0.01<br>0.01<br>0.09<br>0.002	0.01<br>0.01<br>0.06<br>0.003	 1.53<br>1.48<br>2.81	 0.05<br>0.15<br>0.03	 0.05<br>0.16<br>0.02
**rs9837024**	Chr 3,		GWAS	G/A	0.36/0.22	Allelic	1.99	0.0002	0.0003			

	78,534,327					Additive	2.09	0.0002	0.0002	1.95	0.002	0.001
					GWAS	Dominant	2.01	0.006	0.006	2.06	0.008	0.008
					0.36/0.21	Recessive	4.51	0.0001	0.001	3.27	0.10	0.009
rs501833	Chr 3,	*LAMP3*	Additional	C/G	0.13/0.11	Allelic	1.22	0.46	0.46			
	184,360,620					Additive	1.23	0.45	0.44	1.10	0.74	0.76
						Dominant	1.21	0.51	0.52	1.11	0.75	0.75
						Recessive	2.02	0.52	0.35	1.20	0.88	0.44
rs500910	Chr 3,	*LAMP3*	GWAS	A/T	0.19/0.32	Allelic	0.51	0.002	0.002			
	184,360,726					Additive	0.51	0.002	0.002	0.52	0.005	0.007
					GWAS	Dominant	0.41	0.0007	0.0004	0.43	0.003	0.001
					0.20/0.37	Recessive	0.59	0.32	0.29	0.53	0.26	0.24
rs1965484	Chr 3,	*LAMP3*	GWAS	A/C	0.32/0.42	Allelic	0.64	0.02	0.02			
	184,361,591					Additive	0.64	0.02	0.01	0.63	0.02	0.02
					GWAS	Dominant	0.46	0.002	0.002	0.45	0.004	0.003
					0.32/0.48	Recessive	0.87	0.67	0.72	0.75	0.44	0.44
rs522838	Chr 3,	*LAMP3*	Additional	G/C	0.13/0.21	Allelic	0.57	0.02	0.03			
	184,365,224					Additive	0.57	0.03	0.02	0.58	0.04	0.04
						Dominant	0.55	0.03	0.04	0.56	0.06	0.05
						Recessive	0.30	0.23	0.15	0.28	0.22	0.15
rs17597256	Chr 3,	*DLG1*	GWAS	T/C	0.46/0.40	Allelic	1.27	0.21	0.22			
	198,252,251					Additive	1.26	0.23	0.22	1.11	0.60	0.60
					GWAS	Dominant	0.90	0.69	0.70	0.79	0.44	0.45
					0.47/0.35	Recessive	2.25	0.007	0.005	1.87	0.06	0.05
**rs4242051**	Chr 5,		GWAS	T/C	0.40/0.28	Allelic	1.71	0.003	0.005			
	54,234,532					Additive	1.68	0.004	0.003	1.41	0.08	0.08
					GWAS	Dominant	1.47	0.13	0.11	1.16	0.59	0.59
					0.38/0.26	Recessive	3.30	0.0002	0.0004	2.73	0.005	0.004
rs10223133^	Chr5,	*RASGRF2*	Additional	A/G	0.18/0.20	Allelic	0.86	0.51	0.52			
	80,469,216					Additive	0.85	0.49	0.52	0.89	0.64	0.65
						Dominant	0.81	0.42	0.41	0.82	0.50	0.51
						Recessive	1.12	0.88	0.86	1.54	0.60	0.46
rs248989^	Chr 5,	*RASGRF2*	Additional	C/T	0.54/0.44	Allelic	1.53	0.01	0.02			
	80,472,340					Additive	1.50	0.02	0.02	1.43	0.05	0.05
						Dominant	1.72	0.07	0.07	1.84	0.06	0.06
						Recessive	1.75	0.04	0.05	1.50	0.18	0.17
rs17213955^	Chr 5,	*RASGRF2*	Additional	T/C	0.18/0.24	Allelic	0.69	0.10	0.09			
	80,472,997					Additive	0.67	0.09	0.09	0.69	0.14	0.15
						Dominant	0.64	0.09	0.09	0.66	0.15	0.16
						Recessive	0.59	0.47	0.48	0.58	0.49	0.54
rs369844^	Chr 5,	*RASGRF2*	Additional	T/C	0.25/0.29	Allelic	0.83	0.35	0.35			
	80,483,382					Additive	0.82	0.33	0.34	0.78	0.27	0.27
						Dominant	0.83	0.46	0.49	0.80	0.41	0.42
						Recessive	0.57	0.36	0.42	0.51	0.29	0.32
rs4836493	Chr 5,	*CSS3*	GWAS	A/G	0.11/0.07	Allelic	1.69	0.06	0.07			
	129,499,621					Additive	1.68	0.07	0.05	1.87	0.04	0.04

					GWAS<br>0.13/0.04	Dominant<br>Recessive	1.73<br>2.54	0.08<br>0.41	0.06<br>0.17	1.95<br>2.84	0.05<br>0.38	0.05<br>0.13
rs1017213	Chr 5,<br>163,281,156		GWAS	T/C	0.44/0.31<br><br>GWAS<br>0.46/0.29	Allelic<br>Additive<br>Dominant<br>Recessive	1.77<br>1.70<br>2.00<br>2.14	0.001<br>0.002<br>0.009<br>0.02	0.002<br>0.0008<br>0.009<br>0.009	 1.54<br>1.83<br>1.70	 0.02<br>0.03<br>0.14	 0.02<br>0.03<br>0.12
**rs6459959**	Chr 7,<br>155,390,912		GWAS	C/G	0.51/0.36<br><br>GWAS<br>0.47/0.33	Allelic<br>Additive<br>Dominant<br>Recessive	1.89<br>1.88<br>2.04<br>2.66	0.0003<br>0.0004<br>0.01<br>0.0007	0.0003<br>0.001<br>0.01<br>0.0008	 1.78<br>2.08<br>2.30	 0.002<br>0.02<br>0.008	 0.003<br>0.02<br>0.005
**rs6459961**	Chr 7,<br>155,391,016		GWAS	C/A	0.51/0.36<br><br>GWAS<br>0.49/0.32	Allelic<br>Additive<br>Dominant<br>Recessive	1.89<br>1.88<br>2.04<br>2.66	0.0003<br>0.0004<br>0.01<br>0.0007	0.0003<br>0.001<br>0.01<br>0.0008	 1.78<br>2.07<br>2.30	 0.002<br>0.02<br>0.008	 0.003<br>0.01<br>0.005
**rs10104640**	Chr 8,<br>40,762,563	*ZMAT4*	GWAS	A/C	0.38/0.27<br><br>GWAS<br>0.37/0.25	Allelic<br>Additive<br>Dominant<br>Recessive	1.66<br>1.58<br>1.29<br>3.43	0.006<br>0.009<br>0.32<br>6.7X10^{-5}	0.007<br>0.007<br>0.31<br>0.0002	 1.63<br>1.39<br>3.51	 0.01<br>0.23<br>0.0003	 0.007<br>0.23<br>0.0004
rs4736880	Chr 8,<br>40,772,063	*ZMAT4*	Additional	T/G	0.56/0.46	Allelic<br>Additive<br>Dominant<br>Recessive	1.48<br>1.44<br>1.19<br>2.16	0.03<br>0.03<br>0.54<br>0.003	0.04<br>0.04<br>0.49<br>0.003	 1.51<br>1.22<br>2.38	 0.03<br>0.51<br>0.003	 0.03<br>0.51<br>0.002
rs11782961[@]	Chr 8,<br>124,369,839		r^2=0.96<br>with<br>GWAS<br>SNP<br>rs1044714	A/G	0.37/0.23<br>GWAS<br>rs1044714<br>0.37/0.22	Allelic<br>Additive<br>Dominant<br>Recessive	1.97<br>1.91<br>1.85<br>3.78	0.0002<br>0.0004<br>0.01<br>0.0001	0.0006<br>0.0008<br>0.01<br>0.0006	 1.87<br>1.98<br>3.26	 0.001<br>0.01<br>0.003	 0.001<br>0.01<br>0.003
rs6470145	Chr 8,<br>124,391,920		Additional	A/G	0.14/0.07	Allelic<br>Additive<br>Dominant<br>Recessive	2.11<br>2.09<br>2.23<br>3.07	0.008<br>0.01<br>0.01<br>0.32	0.006<br>0.007<br>0.006<br>0.06	 1.83<br>1.83<br>4.46	 0.06<br>0.09<br>0.19	 0.06<br>0.09<br>0.07
rs6477941	Chr 9,<br>114,292,170	*KIAA1958*	GWAS	T/C	0.12/0.05<br><br>GWAS<br>0.12/0.04	Allelic<br>Additive<br>Dominant<br>Recessive	2.46<br>2.40<br>2.60<br>3.39	0.001<br>0.002<br>0.002<br>0.29	0.003<br>0.0008<br>0.0008<br>0.18	 2.20<br>2.27<br>4.72	 0.01<br>0.02<br>0.22	 0.01<br>0.01<br>0.05
rs10857508	Chr 10,<br>50,443,270		GWAS	C/G	0.14/0.10<br><br>GWAS<br>0.16/0.09	Allelic<br>Additive<br>Dominant<br>Recessive	1.54<br>1.49<br>1.21<br>6.70	0.10<br>0.11<br>0.54<br>0.001	0.12<br>0.11<br>0.60<br>0.005	 1.48<br>1.30<br>4.73	 0.13<br>0.43<br>0.02	 0.13<br>0.42<br>0.02
rs10832607	Chr 11,<br>16,419,343	*SOX6*	GWAS	T/C	0.28/0.19<br><br>GWAS<br>0.29/0.20	Allelic<br>Additive<br>Dominant<br>Recessive	1.69<br>1.68<br>1.30<br>6.06	0.007<br>0.009<br>0.30<br>5.3X10^{-6}	0.01<br>0.007<br>0.31<br>0.0002	 1.81<br>1.40<br>7.66	 0.006<br>0.22<br>1.2X10^{-5}	 0.006<br>0.24<br>0.0002
**rs10500830**	Chr 11,<br>16,441,827	*SOX6*	GWAS	A/G	0.28/0.19	Allelic<br>Additive	1.71<br>1.71	0.006<br>0.007	0.008<br>0.007	 1.83	 0.005	 0.005

					GWAS<br>0.29/0.20	Dominant<br>Recessive	1.40<br>5.37`	0.18<br>$3.6X10^{-5}$	0.17<br>0.0002	1.54<br>6.18	0.12<br>0.0001	0.13<br>0.0002
**rs12279572**	Chr 11,<br>117,458,170	*TMPRSS4*	GWAS	G/A	0.36/0.29<br><br>GWAS<br>0.36/0.28	Allelic<br>Additive<br>Dominant<br>Recessive	1.39<br>1.37<br>0.94<br>3.61	0.07<br>0.08<br>0.79<br>$3.5X10^{-5}$	0.08<br>0.08<br>0.78<br>0.0004	 1.44<br>1.06<br>3.61	 0.06<br>0.84<br>0.0002	 0.05<br>0.85<br>0.0006
rs10892210	Chr 11,<br>117,461,548	*TMPRSS4*	Additional	T/G	0.44/0.39	Allelic<br>Additive<br>Dominant<br>Recessive	1.24<br>1.23<br>1.15<br>1.59	0.22<br>0.23<br>0.60<br>0.12	0.26<br>0.23<br>0.60<br>0.12	 1.19<br>1.06<br>1.61	 0.35<br>0.84<br>0.14	 0.33<br>0.86<br>0.12
**rs7975290**	Chr 12,<br>26,505,185	*ITPR2*	GWAS	G/A	0.14/0.05<br><br>GWAS<br>0.13/0.05	Allelic<br>Additive<br>Dominant<br>Recessive	2.96<br>2.91<br>3.56<br>-	$2.5X10^{-5}$<br>$8.4X10^{-5}$<br>$1.4X10^{-5}$<br>1.00	0.0004<br>0.0004<br>0.0002<br>0.91	 3.53<br>4.23<br>-	 $4.5X10^{-5}$<br>$1.4X10^{-5}$<br>1.00	 0.0006<br>0.0002<br>0.34
**rs10773689**	Chr 12,<br>128,686,079	*TMEM132D*	GWAS	A/C	0.24/0.39<br><br>GWAS<br>0.23/0.41	Allelic<br>Additive<br>Dominant<br>Recessive	0.50<br>0.50<br>0.48<br>0.24	0.0005<br>0.0007<br>0.003<br>0.02	0.001<br>0.0004<br>0.002<br>0.01	 0.47<br>0.45<br>0.19	 0.0005<br>0.004<br>0.008	 0.0008<br>0.004<br>0.003
rs11060480	Chr 12,<br>128,692,468	*TMEM132D*	GWAS	G/A	0.65/0.48<br><br>GWAS<br>0.34/0.48	Allelic<br>Additive<br>Dominant<br>Recessive	1.96<br>1.99<br>3.26<br>2.17	0.0002<br>0.0002<br>0.004<br>0.003	0.0003<br>0.0004<br>0.004<br>0.003	 2.23<br>3.59<br>2.51	 0.0001<br>0.003<br>0.002	 0.0004<br>0.003<br>0.001
**rs10519861**	Chr 15,<br>31,773,350	*RYR3*	GWAS	T/G	0.56/0.40<br><br>GWAS<br>0.44/0.39	Allelic<br>Additive<br>Dominant<br>Recessive	1.96<br>2.00<br>2.74<br>2.34	0.0001<br>0.0001<br>0.002<br>0.002	<0.0001<br>0.0002<br>0.0006<br>0.003	 2.18<br>2.90<br>2.67	 0.0001<br>0.002<br>0.002	 0.0002<br>0.002<br>0.002
**rs8027394**	Chr 15,<br>76,404,968		GWAS	T/C	0.48/0.30<br><br>GWAS<br>0.47/0.31	Allelic<br>Additive<br>Dominant<br>Recessive	2.12<br>2.06<br>2.49<br>2.86	$1.8X10^{-5}$<br>$4.2X10^{-5}$<br>0.0009<br>0.0006	<0.0001<br>0.0002<br>0.001<br>0.001	 2.12<br>2.24<br>3.44	 0.0002<br>0.006<br>0.0004	 0.0004<br>0.006<br>0.0008
rs925987	Chr 15,<br>76,406,948		Additional	C/T	0.49/0.33	Allelic<br>Additive<br>Dominant<br>Recessive	1.96<br>1.93<br>2.26<br>2.62	0.0001<br>0.0002<br>0.003<br>0.001	0.0002<br>0.001<br>0.003<br>0.001	 1.91<br>1.98<br>2.88	 0.001<br>0.02<br>0.002	 0.001<br>0.02<br>0.003
**rs12593362**	Chr 15,<br>76,421,786	*CRABP1*	Additional	T/G	0.47/0.31	Allelic<br>Additive<br>Dominant<br>Recessive	1.99<br>1.99<br>2.70<br>2.26	$8.4X10^{-5}$<br>0.0001<br>0.0004<br>0.01	<0.0001<br>0.0002<br>0.0004<br>0.005	 2.10<br>2.82<br>2.40	 0.0002<br>0.0007<br>0.02	 0.0004<br>0.0006<br>0.01
rs2437788	Chr 15,<br>84,987,337		Additional	T/C	0.44/0.38	Allelic<br>Additive<br>Dominant<br>Recessive	1.26<br>1.25<br>1.42<br>1.26	0.19<br>0.20<br>0.19<br>0.48	0.20<br>0.19<br>0.19<br>0.42	 1.23<br>1.48<br>1.10	 0.28<br>0.17<br>0.79	 0.27<br>0.17<br>0.82
rs2469182[±]	Chr 15,<br>84,990,324		r2=0.86<br>with<br>GWAS	A/T	0.18/0.10<br>GWAS<br>rs7183919	Allelic<br>Additive<br>Dominant	1.93<br>1.88<br>2.14	0.004<br>0.006<br>0.005	0.006<br>0.004<br>0.002	 1.81<br>2.06	 0.02<br>0.01	 0.02<br>0.01

			SNP<br>rs7183919		0.22/0.11	Recessive	1.86	0.43	0.17	1.66	0.56	0.35
**rs8058644**	Chr 16,<br>48,454,181		GWAS	T/C	0.18/0.07<br><br>GWAS<br>0.16/0.06	Allelic<br>Additive<br>Dominant<br>Recessive	2.72<br>2.61<br>3.02<br>3.42	$1.9X10^{-5}$<br>$6.8X10^{-5}$<br>$6.3X10^{-5}$<br>0.14	0.0002<br>0.0004<br>0.0004<br>0.03	 2.86<br>3.50<br>3.33	 $8.7X10^{-5}$<br>$5.8X10^{-5}$<br>0.17	 0.0004<br>0.0006<br>0.13
rs17212151	Chr 16,<br>48,472,935		Additional	T/C	0.18/0.10	Allelic<br>Additive<br>Dominant<br>Recessive	2.03<br>1.96<br>2.24<br>2.04	0.002<br>0.004<br>0.003<br>0.36	0.005<br>0.003<br>0.003<br>0.20	 2.23<br>2.85<br>1.93	 0.001<br>0.0006<br>0.42	 0.0008<br>0.0004<br>0.24
rs12968495	Chr 18,<br>10,267,737		Additional	C/T	0.31/0.36	Allelic<br>Additive<br>Dominant<br>Recessive	0.81<br>0.82<br>0.84<br>0.60	0.27<br>0.28<br>0.48<br>0.25	0.29<br>0.29<br>0.49<br>0.22	 0.90<br>0.87<br>0.86	 0.60<br>0.62<br>0.76	 0.60<br>0.61<br>0.77
**rs16974035**	Chr 18,<br>10,269,356		GWAS	G/A	0.49/0.34<br><br>GWAS<br>0.48/0.31	Allelic<br>Additive<br>Dominant<br>Recessive	1.86<br>1.86<br>2.00<br>2.68	0.0004<br>0.0005<br>0.01<br>0.001	0.0005<br>0.0004<br>0.008<br>0.001	 1.96<br>2.08<br>3.07	 0.0005<br>0.01<br>0.0008	 0.001<br>0.009<br>0.0006
rs9959544	Chr 18,<br>55,478,330	*CCBE1*	Additional	G/A	0.25/0.36	Allelic<br>Additive<br>Dominant<br>Recessive	0.58<br>0.58<br>0.47<br>0.63	0.006<br>0.007<br>0.003<br>0.29	0.008<br>0.006<br>0.002<br>0.28	 0.66<br>0.54<br>0.84	 0.06<br>0.02<br>0.71	 0.06<br>0.02<br>0.70
rs8093323[©]	Chr 18,<br>55,479,651	*CCBE1*	r2=0.84<br>with<br>GWAS<br>SNP<br>rs6567111	G/A	0.25/0.36<br>GWAS<br>rs6567111<br>0.24/0.41	Allelic<br>Additive<br>Dominant<br>Recessive	0.59<br>0.59<br>0.48<br>0.65	0.008<br>0.009<br>0.004<br>0.34	0.01<br>0.007<br>0.003<br>0.29	 0.68<br>0.55<br>0.91	 0.08<br>0.03<br>0.84	 0.08<br>0.03<br>0.84
rs2062980	Chr 18,<br>60,573,886		GWAS	T/G	0.52/0.41<br><br>GWAS<br>0.45/0.38	Allelic<br>Additive<br>Dominant<br>Recessive	1.58<br>1.57<br>1.72<br>1.94	0.009<br>0.01<br>0.06<br>0.02	0.01<br>0.009<br>0.06<br>0.02	 1.43<br>1.46<br>1.77	 0.07<br>0.22<br>0.07	 0.07<br>0.22<br>0.07
rs1011636	Chr 20,<br>50,264,021		Additional	G/C	0.46/0.41	Allelic<br>Additive<br>Dominant<br>Recessive	1.25<br>1.24<br>1.33<br>1.35	0.20<br>0.21<br>0.29<br>0.32	0.22<br>0.20<br>0.31<br>0.28	 1.27<br>1.31<br>1.46	 0.21<br>0.36<br>0.25	 0.21<br>0.36<br>0.25
rs2016367	Chr 20,<br>50,276,712		GWAS	C/G	0.30/0.22<br><br>GWAS<br>0.29/0.20	Allelic<br>Additive<br>Dominant<br>Recessive	1.48<br>1.46<br>1.25<br>3.28	0.04<br>0.05<br>0.37<br>0.002	0.05<br>0.04<br>0.35<br>0.004	 1.40<br>1.13<br>3.64	 0.11<br>0.66<br>0.002	 0.10<br>0.65<br>0.003

Chr = chromosome; GWAS = genome wide association study; MAF = minor allele frequency; MV = multivariable; SNP = single nucleotide polymorphism; VnD = ventricular dysfunction;

* 71 VnD cases are a successfully genotyped subset of the 76 VnD cases in the initial GWAS.

[#] SNPs in bold font are 19 SNPs (from 15 genetic loci) that were selected for replication study efforts in both the Vanderbilt cohort (Supporting Information Table S3) and in new VnD cases from the CABG Genomics cohort (Supporting Information Table S4).

^ rs42430 GWAS lead SNP failed Sequenom genotyping but 4 alternate additional SNPs were genotyped to represent the linkage disequilibrium blocks in the region.

[@] rs1044714 GWAS lead SNP failed Sequenom genotyping but rs11782961 which is in strong linkage disequilibrium with rs1044714 and was genotyped successfully.

[±] rs7183919 GWAS lead SNP failed primer formation for Sequenom genotyping but rs2469182 which is in linkage disequilibrium with rs7183919 was genotyped successfully.

[©] rs6567111 GWAS lead SNP failed primer formation for Sequenom genotyping but rs8093323 which is in linkage disequilibrium with rs6567111 was genotyped successfully.
