# Supplementary material for: Genome-Wide Assessment for Genetic Variants Associated with Ventricular Dysfunction after Primary Coronary Artery Bypass Graft Surgery
Source: PLoS One. 2011 Sep 30;6(9):e24593. doi: 10.1371/journal.pone.0024593 (PMC3184087; doi:10.1371/journal.pone.0024593)
Supplement: Table S3 — Vanderbilt cohort replication study results: 17 SNP associations (13 genetic loci) with ventricular dysfunction after primary coronary artery bypass graft surgery in 337 European ancestry men. (DOC) [file pone.0024593.s006.doc]

**Supporting Information Table S3. *Vanderbilt cohort replication study results:***

***17 SNP associations (13 genetic loci) with ventricular dysfunction after primary coronary artery bypass graft surgery in 337 European ancestry men***.

| **SNP** | **Chromosome #, Location** | **Minor Allele/ Major Allele** | **MAF VnD Cases (n=58)/ MAF Controls (n=279)** | **Genetic Model*** | **Univariate Odds Ratio** | **Univariate Permuted Point-wise P value** | **Multivariable Adjusted Odds Ratio** | **Multivariable Permuted Point-wise P value** | **Gene** |
| --- | --- | --- | --- | --- | --- | --- | --- | --- | --- |
| **rs1287820** | Chr 1, 180,855,954 | G/C | 14.3%/22.3% | Additive | 0.61 | 0.08 | 0.69 | 0.22 |  |
| **rs17691914** | Chr 3, 34,937,807 | G/A | 12.9%/9.6% | Additive | 1.45 | 0.27 | 1.51 | 0.26 |  |
| **rs9835451** | Chr3, 34,946,568 | G/A | 19.0%/13.8% | Additive | 1.47 | 0.15 | 1.39 | 0.28 |  |
| **rs17358517** | Chr3, 59,660,772 | T/C | 25.9%/23.3% | Additive | 1.15 | 0.63 | 1.28 | 0.35 |  |
| **rs17061085** | Chr3, 59,666,765 | A/G | 17.2%/10.1% | Additive | 1.65 | 0.04 | 1.69 | 0.056 |  |
| **rs9837024** | Chr3, 78,534,327 | Failed genotyping |  |  |  |  |  |  |  |
| **rs4242051** | Chr5, 54,234,532 | T/C | 21.6%/23.7% | Recessive | 0.29 | 0.22 | 0.13 | 0.04 |  |
| **rs6459959** | Chr7, 155,390,912 | C/G | 37.1%/35.0% | Additive | 1.09 | 0.68 | 1.11 | 0.67 |  |
| **rs6459961** | Chr7, 155,391,016 | C/A | 37.1%/34.6% | Additive | 1.12 | 0.64 | 1.14 | 0.60 |  |
| **rs10104640** | Chr8, 40,762,563 | A/C | 21.9%/23.5% | Recessive | - | 0.96 | - | 0.88 | *ZMAT4* |
| **rs10500830** | Chr11, 16,441,827 | A/G | 19.8%/21.5% | Recessive | 0.63 | 0.60 | 0.57 | 0.38 | *SOX6* |
| **rs12279572** | Chr11, 117,458,170 | G/A | 35.3%/30.1% | Recessive | 1.86 | 0.17 | 2.14 | 0.10 | *TMPRSS4* |
| **rs7975290** | Chr12, 26,505,185 | G/A | 3.4%/5.2% | Additive | 0.65 | 0.41 | 0.71 | 0.57 | *ITPR2* |
| **rs10773689** | Chr12, 128,686,079 | Controls not in HWE^#^ |  |  |  |  |  |  | *TMEM132D* |
| **rs10519861** | Chr15, 31,773,350 | T/G | 46.6%/39.8% | Additive | 1.30 | 0.20 | 1.38 | 0.16 | *RYR3* |
| **rs8027394** | Chr15, 76,404,968 | T/C | 28.5%/28.5% | Additive | 1.00 | 0.93 | 0.91 | 0.68 |  |
| **rs12593362** | Chr15, 76,421,786 | T/G | 31.6%/30.3% | Additive | 1.06 | 0.80 | 1.08 | 0.76 |  |
| **rs8058644** | Chr16, 48,454,181 | T/C | 6.0%/5.7% | Additive | 1.06 | 0.97 | 1.09 | 0.91 |  |
| **rs16974035** | Chr18, 10,269,356 | G/A | 29.8%/35.0% | Additive | 0.81 | 0.33 | 0.87 | 0.55 |  |

***** results using best genetic model from genome wide association study (additive, dominant, or recessive models)

^#^ HWE = Hardy Weinberg equilibrium

MAF = minor allele frequency; SNP = single nucleotide polymorphism; VnD = ventricular dysfunction
