# Supplementary material for: Genome-Wide Assessment for Genetic Variants Associated with Ventricular Dysfunction after Primary Coronary Artery Bypass Graft Surgery
Source: PLoS One. 2011 Sep 30;6(9):e24593. doi: 10.1371/journal.pone.0024593 (PMC3184087; doi:10.1371/journal.pone.0024593)
Supplement: Table S4 — CABG Genomics cohort replication study results: 19 SNP associations (15 genetic loci) with ventricular dysfunction after primary coronary artery bypass graft surgery in 980 European ancestry men. (DOC) [file pone.0024593.s007.doc]

**Supporting Information Table S4*. CABG Genomics cohort replication study results:***

***19 SNP associations (15 genetic loci) with ventricular dysfunction after primary coronary artery bypass graft surgery in 980 European ancestry men.***

| **SNP** | **Chromosome #, Location** | **Minor Allele/ Major Allele** | **MAF VnD Cases (n=59)/ MAF Controls (n=921)** | **Genetic Model*** | **Univariate Odds Ratio** | **Univariate Permuted Point-wise P value** | **Multivariable Adjusted Odds Ratio** | **Multivariable Permuted Point-wise P value** | **Gene** |
| --- | --- | --- | --- | --- | --- | --- | --- | --- | --- |
| **rs1287820** | Chr1, 180,855,954 | G/C | 19.5%/20.6% | Additive | 0.93 | 0.79 | 1.02 | 0.93 |  |
| **rs17691914** | Chr3, 34,937,807 | G/A | 8.5%/7.8% | Additive | 1.10 | 0.77 | 1.25 | 0.52 |  |
| **rs9835451** | Chr3, 34,946,568 | G/A | 13.6%/11.8% | Additive | 1.17 | 0.67 | 1.17 | 0.58 |  |
| **rs17358517** | Chr3, 59,660,772 | T/C | 22.0%/22.5% | Additive | 0.98 | 0.98 | 1.04 | 0.85 |  |
| **rs17061085** | Chr3, 59,666,765 | A/G | 16.1%/12.7% | Additive | 1.25 | 0.31 | 1.29 | 0.26 |  |
| **rs9837024** | Chr3, 78,534,327 | G/A | 22.0%/21.6% | Additive | 1.03 | 0.90 | 0.98 | 0.92 |  |
| **rs4242051** | Chr5, 54,234,532 | T/C | 25.4%/27.8% | Recessive | 1.06 | 0.86 | 1.02 | 0.96 |  |
| **rs6459959** | Chr7, 155,390,912 | C/G | 41.5%/36.0% | Additive | 1.27 | 0.21 | 1.26 | 0.25 |  |
|  |  |  |  | Dominant | 1.72 | 0.07 | 1.75 | 0.06 |  |
| **rs6459961** | Chr7, 155,391,016 | C/A | 41.5%/36.0% | Additive | 1.27 | 0.23 | 1.26 | 0.24 |  |
|  |  |  |  | Dominant | 1.73 | 0.07 | 1.75 | 0.06 |  |
| **rs10104640** | Chr8, 40,762,563 | A/C | 26.3%/27.2% | Recessive | 1.04 | 0.88 | 0.95 | 0.96 | *ZMAT4* |
| **rs10500830** | Chr11, 16,441,827 | A/G | 19.5%/19.1% | Recessive | 1.64 | 0.17 | 1.95 | 0.10 | *SOX6* |
| **rs12279572** | Chr11, 117,458,170 | G/A | 24.6%/28.8% | Recessive | 1.07 | 0.96 | 1.00 | 0.99 | *TMPRSS4* |
| **rs7975290** | Chr12, 26,505,185 | G/A | 2.5%/5.2% | Additive | 0.49 | 0.27 | 0.49 | 0.23 | *ITPR2* |
| **rs10773689** | Chr12, 128,686,079 | A/C | 40.7%/39.7% | Additive | 1.04 | 0.80 | 1.01 | 0.96 | *TMEM132D* |
| **rs10519861** | Chr15, 31,773,350 | T/G | 35.6%/39.4% | Additive | 0.85 | 0.38 | 0.80 | 0.27 | *RYR3* |
| **rs8027394** | Chr15, 76,404,968 | T/C | 22.9%/31.0% | Additive | 0.67 | 0.08 | 0.62 | 0.04 |  |
| **rs12593362** | Chr15, 76,421,786 | T/G | 31.4%/31.5% | Additive | 0.99 | 0.97 | 0.97 | 0.88 |  |
| **rs8058644** | Chr16, 48,454,181 | T/C | 7.6%/7.3% | Additive | 1.04 | 0.95 | 1.13 | 0.72 |  |
| **rs16974035** | Chr18, 10,269,356 | G/A | 39.8%/33.2% | Additive | 1.33 | 0.15 | 1.42 | 0.08 |  |

***** results using best genetic model from genome wide association study (additive, dominant, or recessive models)

MAF = minor allele frequency; SNP = single nucleotide polymorphism; VnD = ventricular dysfunction
