# Supplementary material for: Genome-Wide Assessment for Genetic Variants Associated with Ventricular Dysfunction after Primary Coronary Artery Bypass Graft Surgery
Source: PLoS One. 2011 Sep 30;6(9):e24593. doi: 10.1371/journal.pone.0024593 (PMC3184087; doi:10.1371/journal.pone.0024593)
Supplement: Table S5 — Covariate adjusted meta-analysis results: 17 SNP associations (13 genetic loci) with ventricular dysfunction after primary coronary artery bypass graft surgery in 1388 European ancestry men. (DOC) [file pone.0024593.s008.doc]

**Supporting Information Table S5*. Covariate adjusted meta-analysis results:****

17 SNP associations (13 genetic loci) with ventricular dysfunction after primary coronary artery bypass graft surgery in 1388 European ancestry men.

| **SNP** | **MAF VnD Cases (n=188)/ MAF Controls (n=1200)** | **Genetic Model** ^†^ | **Odds Ratio** | **P value** | **Heterogeneity P value**^‡^ | **I^2^ Index of Heterogeneity (0-100%)** |
| --- | --- | --- | --- | --- | --- | --- |
| **rs1287820** | 22.9%/21.0% | Additive | 0.99 | 0.98 | 0.01 | 77 |
| **rs17691914** | 13.8%/8.2% | Additive | 2.01 | 0.0002 | 0.64 | 0 |
|  |  | Dominant | 2.20 | 8.1X10^-5^ | 0.50 | 0 |
| **rs9835451** | 18.4%/12.2% | Additive | 1.61 | 0.003 | 0.80 | 0 |
| **rs17358517** | 29.3%/22.7% | Additive | 1.46 | 0.02 | 0.29 | 19 |
| **rs17061085** | 20.2%/12.0% | Additive | 1.70 | 0.0001 | 0.72 | 0 |
| **rs4242051** | 29.8%/26.8% | Recessive | 1.30 | 0.67 | 0.07 | 62 |
| **rs6459959** | 43.9%/35.7% | Additive | 1.41 | 0.005 | 0.49 | 0 |
| **rs6459961** | 43.9%/35.6% | Additive | 1.42 | 0.004 | 0.54 | 0 |
| **rs10104640** | 29.5%/26.3% | Recessive | 2.25 | 0.03 | 0.31 | 14 |
| **rs10500830** | 22.9%/19.7% | Recessive | 2.21 | 0.21 | 0.08 | 60 |
| **rs12279572** | 32.2%/29.1% | Recessive | 2.19 | 0.001 | 0.61 | 0 |
| **rs7975290** | 7.2%/ 5.2% | Additive | 1.83 | 0.37 | 0.004 | 82 |
| **rs10519861** | 46.8%/39.5% | Additive | 1.36 | 0.01 | 0.81 | 0 |
| **rs8027394** | 34.0%/30.4% | Additive | 1.12 | 0.38 | 0.56 | 0 |
| **rs12593362** | 37.4%/31.2% | Additive | 1.30 | 0.03 | 0.58 | 0 |
| **rs8058644** | 10.9%/ 7.0% | Additive | 1.80 | 0.009 | 0.31 | 15 |
| **rs16974035** | 40.1%/33.6% | Additive | 1.58 | 0.17 | 0.005 | 81 |

* Cases and controls are from the CABG Genomics validation and replication studies and the Vanderbilt replication study. Data for the 17 SNPs assessed in the CABG Genomics validation study and the Vanderbilt and CABG Genomics replication studies were analyzed for association with ventricular dysfunction at each of the three enrolling institutions (with covariate adjustments made for age, preoperative left ventricular ejection fraction, and duration of cardiopulmonary bypass). The institutional covariate adjusted SNP association results were combined using random effects meta-analysis (odds ratios and P values derived using random effects meta-analysis).

^†^ Results for best genetic models in the GWAS (additive, dominant, or recessive)

^‡^ P value for Cochran’s Q statistic assessment of heterogeneity (P<0.05 indicates that effect sizes between institutions are significantly different).

^§^ I^2^ Index of Heterogeneity = 0 means that between institution variability (the excess variation) in effect size estimates is secondary to sampling error within the study institutions. However, an I^2^ index value = 50 indicates that 50% of the total variability in effect sizes is not caused by sampling error within institutions but by heterogeneity between institutions. An I^2^ index value ≤ 25 is thought to signify low heterogeneity between institutions.[1]

**Reference**

1. Higgins JP, Thompson SG (2002) Quantifying heterogeneity in a meta-analysis. Stat Med 21: 1539-1558.
